# Compartment-specific redox modulation in migraine with aura: a systematic review of context-dependent mechanisms

**DOI:** 10.22514/jofph.2026.059

**Published:** 2026-09-12

**Authors:** Fengyu Li, Qishen Duan, Yu Li, Yilang Tian, Lijuan Lu, Xin Xin, Guoliang Zhu, Ying Zhou, Fanyi Kong

**Affiliations:** ^1^Department of Neurology, The Affiliated Hospital of Yunnan University, 650021 Kunming, Yunnan, China; ^2^School of Clinical Medicine, Kunming Medical University, 650500 Kunming, Yunnan, China; ^3^School of Clinical Medicine, Dali University, 671003 Dali, Yunnan, China; ^4^School of Medicine, University of Electronic Science and Technology of China, 610054 Chengdu, Sichuan, China; ^5^College of Chemical Science and Technology, Yunnan University, 650091 Kunming, Yunnan, China

**Keywords:** Migraine with aura, Cortical spreading depression, Oxidative stress, Microglial polarization, Redox targeting, Systematic review

## Abstract

**Background**: Preventive therapies for migraine with aura (MA) remain suboptimal, with 37–54% of patients failing to achieve a >50% reduction in migraine days despite calcitonin gene-related peptide (CGRP)-targeted therapies, while broad-spectrum antioxidants show inconsistent benefits. This gap suggests that indiscriminate reactive oxygen species (ROS) scavenging is insufficient, but emerging evidence indicates that ROS function as compartment-specific signaling molecules rather than uniform toxins. Whether this compartment-specific redox modulation occurs within the cortical spreading depression (CSD) cascade and contributes to the limited efficacy of antioxidant therapies remains unclear. We synthesized preclinical evidence to characterize CSD–redox interactions and identify the translational gap limiting precision strategies for MA. **Methods**: We systematically searched five databases through June 2025 following the Preferred Reporting Items for Systematic Reviews and Meta-Analyses (PRISMA) 2020 guidelines. Risk of bias was assessed using the Systematic Review Centre for Laboratory Animal Experimentation (SYRCLE) risk-of-bias tool. Given the anticipated heterogeneity, a qualitative synthesis was conducted. **Results**: Thirteen studies (594 animals, 85% male) were included. CSD-evoked ROS were associated with transient receptor potential ankyrin 1 (TRPA1)/CGRP signaling and oxidative wavefront propagation. Under bioenergetic stress, ROS accumulation was associated with metabolic collapse, which may trigger CSD onset. Microglial M1-like polarization was associated with reduced CSD thresholds. High-dose melatonin accelerated CSD propagation without improving oxidative markers, suggesting limitations of global antioxidant strategies rather than invalidation of the redox framework. Cell-specific protective mechanisms emerged through microglial M2a-like polarization and compartment-specific stabilization of insulin-like growth factor-1 (IGF-1). **Conclusion**: CSD–redox interactions represent a context-dependent reciprocal axis. Clinical failure of broad-spectrum scavengers may reflect disruption of oxidative eustress. Modulators of microglial M2a-like phenotype and compartment-specific bioenergetic stabilizers should be prioritized for future drug development. Intranasal IGF-1 represents a potentially translatable strategy for attenuating trigeminal CGRP release and oxidative markers, although this requires validation in female cohorts and assessment of sex-specific effects. **The PROSPERO Registration**: CRD420251143073.

## 1. Introduction

Migraine is among the leading causes of disability worldwide [1], with approximately 30% of patients suffering from migraine with aura (MA) [2]. In this migraine subtype, cortical spreading depression (CSD) is the widely accepted yet still debated electrophysiological substrate of aura symptoms [3, 4, 5]. Nevertheless, CSD remains one of the most robust preclinical correlates available for understanding the pathophysiology of MA. Because the existence of “silent CSD” in humans remains unproven and the contribution of CSD to migraine without aura is not yet mechanistically resolved, the phenotypic boundaries of any redox framework incorporating CSD must first be specified. Accordingly, we explicitly restrict the proposed redox framework to the MA phenotype and do not extrapolate it to other migraine subtypes [6].

The use of calcitonin gene-related peptide (CGRP) pathway inhibitors has transformed migraine prevention strategies; however, a real-world cohort study reported that approximately 46% of patients who received anti-CGRP monoclonal antibodies failed to achieve a ≥50% reduction in monthly migraine days after 3 months [7]. Notably, broad-spectrum antioxidant interventions, including coenzyme Q10 (CoQ10), vitamin E, and various polyphenolic compounds, have demonstrated modest efficacy in randomized controlled trials [8]. This persistent therapeutic gap suggests that the indiscriminate scavenging of reactive oxygen species (ROS) disrupts physiological redox signaling (oxidative eustress) without resolving compartment-specific pathological foci [9].

CSD constitutes the core electrophysiological event in MA, and its metabolic consequences offer a natural model for understanding this compartment-specific dysregulation. CSD imposes substantial metabolic demands and triggers ionic fluxes that overwhelm adenosine triphosphate (ATP)-dependent pumps. This pump failure induces acute bioenergetic stress, mitochondrial dysfunction and ROS generation [10, 11]. Neuroimaging (magnetoencephalography (MEG)/functional magnetic resonance imaging (fMRI)) has been used to characterize the brain as dysexcitable during migraine [12], with fluctuating cortical responsiveness underpinned by neuronal dysregulation [5] and redox dyshomeostasis [10].

However, this metabolic perspective remains incomplete. Evidence suggests that ROS function not only as downstream byproducts but also as active participants in a feedforward loop that modulates cortical excitability [13]. Preclinical studies suggest that ROS can act as triggers [14] or modulators [15] of CSD susceptibility through transient receptor potential ankyrin 1 (TRPA1) activation [15] or, under bioenergetic stress conditions, through ROS-dependent metabolic collapse (MC) [14].

While preclinical models consistently demonstrate redox disturbances following CSD, attempts to mitigate CSD vulnerability using broad-spectrum antioxidants have yielded inconsistent or biphasic results, including accelerated CSD propagation at high doses [16, 17, 18].

This translational gap suggests that the relationship between CSD and redox homeostasis extends beyond unidirectional oxidative damage to encompass context-dependent reciprocal modulation, wherein ROS may be understood as both consequences and potential triggers depending on the underlying bioenergetic state. Clarifying these dynamics, specifically within the context of the MA phenotype, is essential for understanding the limitations of nonspecific antioxidant strategies. Thus, the development of future precision strategies may involve compartment-specific redox modulation reconciled from preclinical findings and clinical observations. However, human brain redox imaging remains technically challenging [19] and is characterized by substantial marker heterogeneity [16, 17], indicating that animal models remain important for understanding the CSD–redox loop.

Although numerous studies have suggested that CSD disturbs redox homeostasis, preclinical studies have not been systematically synthesized to distinguish physiological redox signaling from pathological damage. Most investigations have considered ROS as uniform downstream products of depolarization; however, the inconsistent or even biphasic effects of broad-spectrum antioxidants cannot be explained under this assumption [18]. Furthermore, a framework that accounts for the cellular source, temporal dynamics, and bioenergetic context of ROS is lacking, leaving the translational potential of redox modulation in MA insufficiently defined. Therefore, the present systematic review integrates preclinical evidence to characterize bidirectional CSD–redox interactions, address the potential mechanisms underlying the clinical failure of antioxidants, and identify potential therapeutic targets for further investigation in MA.

## 2. Methods

### 2.1 Study registration and protocol

This review was conducted in accordance with the PRISMA 2020 statement (**Supplementary material 1**) [20]. We developed a protocol specifying the objectives, search strategy, inclusion criteria, and analysis plan a priori and registered it with the International Prospective Register of Systematic Reviews (PROSPERO; CRD420251143073).

### 2.2 Eligibility criteria

We included animal studies investigating CSD events relevant to MA and their association with redox pathways without species or age restrictions; sex was prespecified as a sensitivity variable to address potential confounding effects on ROS levels [21]. Eligible studies met the following two criteria: (ⅰ) used any method to induce CSD and (ⅱ) evaluated oxidative stress (OS) markers. OS was defined according to Sies [9] as an alteration in redox homeostasis encompassing both physiological signaling (oxidative eustress) and pathological damage (oxidative distress). Reviews, conference abstracts, comments, duplicate data, and studies that did not comply with animal welfare guidelines were excluded. Specifically, studies that did not meet the Animal Research: Reporting of *In Vivo* Experiments (ARRIVE) 2.0 reporting guidelines were excluded from the synthesis.

### 2.3 Literature search strategy

A systematic search was conducted across five databases (MEDLINE via PubMed, Embase, Web of Science, CNKI, and Wanfang) from database inception through June 2025. We combined medical subject headings (MeSH) and free-text keywords to capture four domains: CSD, OS, MA, and rodent models. Citations were deduplicated using EndNote 21.0.1 (Clarivate Analytics, Philadelphia, PA, USA) (**Supplementary material 2**). Additional unpublished studies were identified through manual reference searches and expert consultation.

### 2.4 Study selection and data extraction

Two reviewers (Li and Duan) independently screened the titles and abstracts, assessed the full texts, and extracted data using a standardized form (**Supplementary material 3**). Data were preferentially extracted from the Results section and included (ⅰ) study characteristics; (ⅱ) CSD induction protocols; (ⅲ) redox parameters (categorized into four functional groups, as detailed in the Redox parameter synthesis section) with quantitative values; (ⅳ) bioenergetic indicators, including nicotinamide adenine dinucleotide (phosphate) (NAD(P)H), flavin adenine dinucleotide (FAD), tissue partial pressure of oxygen (pO_2_), reported in a single study [14]; and (ⅴ) functional outcomes (*e.g.*, synaptic recovery). Depolarization events were classified according to the terminology used in the original studies (CSD or spreading depression (SD)); both terms refer to the same CSD phenomenon relevant to MA.

### 2.5 Risk of bias assessment

Two reviewers assessed the risk of bias across ten domains using the SYRCLE tool [22]. Discrepancies were resolved by Kong, and detailed conclusions for all 13 included studies are provided in **Supplementary material 4** (Ref. [13, 14, 15, 18, 23, 24, 25, 26, 27, 28, 29, 30, 31]).

### 2.6 Operational definitions of redox signaling loop components

We defined redox modulation as a change in any of the four parameter groups listed in the Redox parameter synthesis section, thus extending our eligibility criteria for OS to encompass both oxidative shifts and antioxidant responses. We categorized CSD–redox interactions into two functional components based on the following reports in the original study: (ⅰ) forward signaling (CSD-to-redox); *i.e.*, studies reporting statistically significant oxidative shifts (*p* < 0.05) after CSD induction, as measured using redox-sensitive probes or oxidative damage biomarkers; and (ⅱ) reverse signaling (redox-to-CSD); *i.e.*, studies reporting that redox modulation resulted in significant changes in CSD initiation or susceptibility through triggering, mediating, or modulating mechanisms. No quantitative thresholds were prespecified because of the heterogeneity across studies and the limited standardization of redox parameters.

### 2.7 Data synthesis and analysis

The heterogeneity across models, induction protocols, and outcomes precluded meta-analysis. We thus conducted a qualitative synthesis of empirically reported CSD–redox interactions and evaluated heterogeneity based on the variations in study design. The synthesis focused on the temporal sequence of redox shifts, the effects of antioxidant interventions, and calcium ion (Ca^2+^) dependency (bioenergetic data were available for one study only [14]). No predefined mechanistic hypothesis was imposed on the data. Subgroup analyses were prespecified according to the experimental model (*in vivo vs. in vitro*) and species to explore the biological and methodological drivers of heterogeneity. We separately analyzed multiple oxidative endpoints from the same cohort, thereby avoiding aggregation bias while preserving mechanistic insights.

We performed sensitivity analyses to explore robustness by sequentially excluding studies at a high risk of bias or those from a single research group (Kraig’s team [23, 24, 25, 26, 27, 28, 29]). We did not assess publication bias using funnel plots because the synthesis was qualitative. The use of comprehensive searches across multiple databases and unpublished sources was intended to minimize reporting bias.

### 2.8 Redox parameter synthesis

We extracted and categorized redox parameters from the Results section into four functional groups: (ⅰ) reactive species, such as superoxide anion (O_2_^−^) and hydrogen peroxide (H_2_O_2_); (ⅱ) real-time redox indicators, including recombinant redox-sensitive green fluorescent protein (roGFP) oxidation dynamics, CellROX fluorescence, and NAD(P)H/FAD autofluorescence (available in one study only); (ⅲ) biomarkers of oxidative damage and neuroinflammation, including malondialdehyde (MDA), protein carbonyl (PC) content, and tumor necrosis factor-alpha (TNF-α) protein expression; and (ⅳ) redox-regulatory systems, including superoxide dismutase (SOD) activity, glutathione (GSH) levels, and inducible nitric oxide synthase (iNOS) expression.

### 2.9 Certainty assessment

We excluded the Grading of Recommendations Assessment, Development and Evaluations (GRADE) [32] framework because it has not been validated for preclinical research. We instead assessed the certainty of the evidence across four domains: SYRCLE risk of bias, robustness across sensitivity analyses, indirectness of evidence to MA in humans, and precision of the outcomes.

## 3. Results

### 3.1 Characteristics of the included studies

Through database screening, 208 records were identified. Thirteen studies (594 animals) met the inclusion criteria [13, 14, 15, 18, 23, 24, 25, 26, 27, 28, 29, 30, 31] (Fig. 1). In eleven studies (85%), only male animals were used [13, 14, 15, 18, 23, 24, 26, 27, 29, 30, 31]. Mixed or unspecified sexes were reported in P0–10 preparations (*in vitro*) [23, 24, 25, 26, 27, 28]. The rodent models included three species (Table 1, Ref. [13, 14, 15, 18, 23, 24, 25, 26, 27, 28, 29, 30, 31]), with Wistar rats being the predominant model. The studies were published between 2011 and 2024 (Fig. 2). Owing to the substantial methodological heterogeneity across species, induction protocols, and redox detection modalities, we performed a qualitative synthesis of bidirectional modulation patterns rather than a pooled quantitative analysis.

**Fig. 1.** PRISMA flow diagram of the study selection process. CSD: cortical spreading depression.

**Table 1.** Baseline characteristics and methodological determinants of the included studies.

Study reference	Species: sex; total sample number; age; weight	Experimental model: type; induction method; method of anesthesia	Redox parameter(s) evaluated	Sample source (tissue/cells)
Viggiano A *et al*. [30] (2011)	Sprague–Dawley rats: M; n = 15; adult; 250–300 g	*in vivo*; 3 M K^+^; pentobarbital	O_2_^−^; H_2_O_2_; SOD	Neocortex (Layer IV)
Grinberg YY *et al*. [28] (2012)	WIS: mixed; n = N/A; pups (P9), N/A	*in vitro*; Elec; N/A	CellROX	Hippocampus (CA3)
Grinberg YY *et al*. [25] (2013)	WIS: mixed; n = 120; pups (P9–10); N/A	*in vitro*; Elec; N/A	CellROX; PC	Microglia; astrocytes (CA3)
Shatillo A *et al*. [31] (2013)	WIS: M; n = 11; 6 weeks; 133–177 g	*in vivo*; 1 M K^+^; isoflurane/D1 urethane	MDA	Cortex; TG
Pusic KM *et al*. [23] (2014)	WIS: M; n = 45; adult; 300–400 g WIS: mixed; n = N/A; pups (P9–10); N/A	*in vivo*: M; 0.5 M K^+^; Isoflurane *in vitro*: pups; Elec/1 M K^+^; N/A	CellROX; iNOS mRNA; PC; GSH	Microglia (M1/M2; CA3, neocortex)
Malkov A *et al*. [14] (2014)	SW: M; n = N/A; P21–56; N/A SW: M; n = 5; >P50; 29–32 g	*in vivo*; 3 M K^+^; ketamine/xylazine *in vitro*: HFS; glutamate; IOA/pyruvate; N/A	CellROX; pO_2_; NAD(P)H; FAD	N/A
Pusic AD *et al*. [24] (2015a)	WIS: mixed; n = 120; pups (P9–10); N/A WIS: M; n = 17; adult; 300–400 g	*in vivo*: M; 0.5 M K^+^; isoflurane *in vitro*: pups; Elec; N/A	CellROX; GSH; PC	T cells; microglia (CA3, neocortex)
Pusic AD *et al*. [27] (2015b)	WIS: mixed; n = N/A; pups (P0–10); N/A WIS: M; n = N/A; adult; 300–400 g	*in vitro*: pups; Elec; N/A *in vivo*: M; 0.5 M K^+^; isoflurane	CellROX; GSH; PC	Microglia (CA3, neocortex)
Pusic KM *et al*. [26] (2019)	WIS: mixed; n = N/A; pups (P9–10); N/A WIS: M; n = N/A; 10–12 weeks; 300–400 g	*in vivo*: M; 0.5 M K^+^; isoflurane *in vitro*: pups; Elec; N/A	iNOS mRNA; PC	Microglia (M1; hippocampus, neocortex)
Jiang L *et al*. [15] (2019)	SD: M; n = 30; adult; 253.8 ± 40.9 g C57BL/6: M; n = 36; N/A; 21.2 ± 2.7 g	*in vivo*: SD; 2 M K^+^; isoflurane *in vitro*: C57BL/6; 0.5 M K^+^; N/A	MDA	Cortex
Won L *et al*. [29] (2020)	WIS: M; n = 90; adult; 250–450 g	*in vivo*; 0.5 M K^+^; isoflurane	MDA	Cortex; TG
Araújo AO *et al*. [18] (2023)	WIS: M; n = 40; pups (P7–42); N/A	*in vivo*; 2% K^+^; urethane and chloralose	MDA; SOD	Cortex
Ackermann MA *et al*. [13] (2024)	roGFPc: M; n = 65; 5.8 ± 2.9 months; N/A	*in vitro*; hypoxia/FCCP: global perfusion/3 M K^+^; ether/isoflurane	OxD (roGFPc)	Neurons (CA1)

*CA1: cornu ammonis area 1; CA3: cornu ammonis area 3; C57BL/6: C57 black 6 inbred mouse strain; CellROX: fluorogenic probe for the detection of reactive oxygen species; Elec: electrical stimulation; FAD: flavin adenine dinucleotide; FCCP: carbonyl cyanide-4-(trifluoromethoxy)phenylhydrazone; GSH: glutathione; H_2_O_2_: hydrogen peroxide; HFS: high-frequency stimulation; iNOS: inducible nitric oxide synthase; IOA: iodoacetamide; K⁺: potassium ion; M: molar/male; MDA: malondialdehyde; NAD(P)H: nicotinamide adenine dinucleotide (phosphate); N/A: not applicable or not available; O_2_^−^: superoxide anion; OxD: oxidation degree; P: postnatal day; PC: protein carbonyl; pO_2_: partial pressure of oxygen; roGFP: redox-sensitive green fluorescent protein; roGFPc: cytosolic roGFP; SOD: superoxide dismutase; SW: Swiss Webster; TG: trigeminal ganglion; WIS: Wistar; g: gram. Table note: These entries show where oxidative stress markers (MDA/SOD) were assayed, not where the ROS originated. Standard immunohistochemistry and colorimetric assays capture tissue-level redox status but cannot distinguish specific cell types (neurons vs. glial cells) or confirm localized ROS production. As such, these locations reflect the indirect oxidative burden rather than the exact source (Jiang, 2019 [15]; Grinberg, 2013 [25]; Grinberg, 2012 [28]; Won, 2020 [29]).*

**Fig. 2.** Methodological characteristics of the included studies. Summary of the study characteristics. (A) Publication years of the included studies. (B) Animal species and strains. (C) Methods used for CSD induction. (D) Intervention types and mechanistic targets. roGFP: redox-sensitive green fluorescent protein; CSD: cortical spreading depression; C57BL/6: C57 black 6 inbred mouse strain; KCl: potassium chloride; IL: interleukin; IFN: interferon; IGF: insulin-like growth factor.

### 3.2 Experimental models and CSD induction parameters

CSD was evoked chemically through focal cortical superfusion with potassium chloride (KCl; 0.5–3 M) in 11 studies [13, 14, 15, 18, 23, 24, 26, 27, 29, 30, 31], whereas electrical pulses were employed to evoke CSD events in 6 studies [23, 24, 25, 26, 27, 28]. The anesthetic agents used varied across the included studies: isoflurane was used in eight studies [13, 15, 23, 24, 26, 27, 29, 31], urethane was employed in two studies [18, 31], ketamine/xylazine was used in one study [14], and pentobarbital was employed in one study [30].

### 3.3 Risk of bias assessment

The SYRCLE assessment indicated a low risk of bias for seven studies [14, 18, 23, 26, 27, 28, 30] and an unclear risk of bias for six studies [13, 15, 24, 25, 29, 31]. For all 13 studies, the risk of bias associated with random sequence generation and allocation concealment was unclear. The risk associated with blinding of the outcome assessors was considered low risk in one study [29]. Three studies were classified as having a high risk of bias because of reported conflicts of interest [24, 25, 27] (Fig. 3, Ref. [13, 14, 15, 18, 23, 24, 25, 26, 27, 28, 29, 30, 31]).

**Fig. 3.** Risk of bias assessment. (a) Risk of bias plot. (b) Summary of the risk of bias assessment.

### 3.4 Redox marker assessment methods

Redox markers were categorized into two distinct detection modalities: real-time kinetic imaging (seven studies [13, 14, 23, 24, 25, 27, 28]) and endpoint biochemical assays (10 studies [15, 18, 23, 24, 25, 26, 27, 29, 30, 31]). Kinetic methodologies provided high temporal resolution through measurements of CellROX fluorescence, roGFP oxidation dynamics, and NAD(P)H/FAD flux. In contrast, endpoint assays primarily quantified MDA levels, SOD activity, PC levels, and H_2_O_2_ levels. Similarly, six studies relied exclusively on endpoint markers, whereas four studies integrated both modalities to confirm the link between depolarization and OS.

### 3.5 Mechanistic patterns of compartment-specific redox modulation

This synthesis indicates that compartmentalization determines whether CSD–redox interactions function primarily as a signals or triggers (Table 2, Ref. [13, 14, 15, 18, 23, 24, 25, 26, 27, 28, 29, 30, 31]). We organized the evidence into three interpretive tiers: acute oxidative wavefronts, the efficacy gap between global and compartment-specific modulation, and neuroimmune gating via microglial polarization. Collectively, these tiers suggest a self-reinforcing loop wherein redox shifts modulate cortical excitability.

**Table 2.** Bidirectional redox–neuroimmune modulation of spreading depression susceptibility.

Study		Direction	CSD parameter(s)	Intervention category/categories	Key mechanistic insights
I. Forward signaling: CSD-evoked redox changes					
	Viggiano *et al*. [30] (2011)	CSD → Redox	Amp, Waves	CSD induction	CSD → hypermetabolism → ↑SOD (compensatory) → dismutation of O_2_^−^ to H_2_O_2_ → ↓O_2_^−^ + ↑H_2_O_2_
	Shatillo *et al*. [31] (2013)	CSD → Redox	Amp, Dur, Lat, Waves	CSD induction	CSD → ROS (↑MDA) → TRPA1 activation → ↑TG nociceptive firing + CGRP release → Migraine pain
	Pusic *et al*. [24] (2015a)	CSD → Redox	Threshold	CSD induction	CSD (recurrent) → T-cell infiltration & IFN-γ↑ → Microglial TNF-α↑ → OS↑(GSH↓) → nSMase2 activation → MBP degradation
	Jiang *et al*. [15] (2019)	CSD → Redox	Lat, AUC, Waves	CSD induction	CSD → ROS (↑MDA) → TRPA1 activation → CGRP release → ↑CSD susceptibility
	Ackermann *et al*. [13] (2024)	CSD → Redox	Amp, Dur, Waves	CSD induction	SD initiation → ionic homeostasis breakdown → massive Ca^2+^ influx → cytosolic ROS → persistent oxidation (severe metabolic stress: hypoxia/FCCP) *vs.* reversible (capacity preserved: K^+^-SD)
II. Reverse signaling: redox modulation of CSD susceptibility					
II.A Bioenergetic stress and CSD initiation					
	Malkov *et al*. [14] (2014)	Redox → CSD (T)	Occurrence rate	Metabolic manipulation	ROS accumulation↓ → NAD(P)H oxidation/↓pO_2_ (oxidative stress) → MC mimics CSD → Tempol inhibits initiation
	Jiang *et al*. [15] (2019)	Redox → CSD (M)	Lat, AUC, Waves	Pharmacological modulation	H_2_O_2_ (exogenous) → TRPA1 activation (neurons/astrocytes) → CGRP release → ↑CSD susceptibility → Positive feedback loop
II.B Systemic antioxidant effects					
	Araujo, A.D.O. *et al*. [18] (2023)	Redox → CSD (M)	Velocity, Amp, Dur	Pharmacological modulation	MLT-10: MDA↓/SOD↑ → CSD: ↓propagation, ↓amp, ↑dur; MLT-40: No effect on MDA or SOD levels → CSD: ↑propagation, ↑amp, ↓dur
II.C Compartment-specific redox modulation					
	Grinberg *et al*. [25] (2013)	Redox → CSD (M)	Threshold	Neurotrophic factor; microglial modulation	Exogenous IGF-1 → selective microglial ↓OS/↓TNF-α (astrocytes are unaffected) → blockade of TNF-α priming (both upstream and downstream) → interruption of positive feedback loop → SD: ↑threshold, ↓susceptibility; CSD → TNF-α/microglial ROS
	Grinberg *et al*. [28] (2012)	Redox → CSD (M)	Threshold	Neurotrophic factor	IGF-1 → ↑endogenous antioxidant capacity → ↓CSD-induced OS → ↓SD susceptibility
	Won *et al*. [29] (2020)	Redox → CSD (M)	Waves	Neurotrophic factor	Intranasal IGF-1 pretreatment → ↓naive TG CGRP (81%) + ↓CSD-induced TG MDA/CGRP/c-Fos
II.D Microglial polarization and CSD thresholds					
	Pusic *et al*. [23] (2014)	Redox → CSD (M)	Threshold	Microglial modulation	EE/IL-11 → microglial M2a polarization (↑Arg-1, ↑IL-10, ↓iNOS), TNF-α unchanged → ↓PC/↓OS → SD: ↑threshold, ↓susceptibility
	Pusic *et al*. [24] (2015a)	Redox → CSD (M)	Threshold	CSD induction	IFN-γ-induced redox shift (↑OS, ↓GSH) → SD threshold (95%)
	Pusic *et al*. [27] (2015b)	Redox → CSD (M)	Threshold	Microglial modulation	Phasic IFN-γ → microglial M2a polarization (transient M1 → M2a polarization: ↑Arg-1, ↑IL-10), ↑GSH → release of IFN-γ-MG-Exos (miR-219-enriched) → ↑MBP, ↓OS → SD: ↑threshold, ↓susceptibility
	Pusic *et al*. [26] (2019)	Redox → CSD (M)	Threshold	Microglial modulation	IFN-γ-DC-Exos → selective M1 inhibition (↓iNOS, TNF-α unchanged) → ↓PC → SD: ↑threshold, ↓susceptibility

*(M): modulator of CSD threshold/susceptibility; (T): trigger of spontaneous CSD events; →: reported causal relationship or leads to; ↑: increase/elevation/activation; ↓: decrease/reduction/inhibition; Amp: amplitude; Arg-1: arginase-1; AUC: area under the curve; Ca^2+^: calcium ion; CGRP: calcitonin gene-related peptide; c-Fos: cellular FBJ osteosarcoma oncogene; CSD: cortical spreading depression; Dur: duration; EE: environmental enrichment; FCCP: carbonyl cyanide-4-(trifluoromethoxy)phenylhydrazone; GSH: glutathione; H_2_O_2_: hydrogen peroxide; hypoxia: hypoxia-induced spreading depression; IFN-γ: interferon-gamma; IFN-γ-DC-Exos: interferon-gamma-stimulated dendritic cell-derived exosomes; IFN-γ-MG-Exos: interferon-gamma-stimulated microglia-derived exosomes; IGF-1: insulin-like growth factor-1; IL-10: interleukin-10; IL-11: interleukin-11; iNOS: inducible nitric oxide synthase; Lat: latency; M1: M1-polarized microglia; M2a: M2a-polarized microglia; MBP: myelin basic protein; MC: metabolic collapse; MDA: malondialdehyde; miR-219: microRNA-219; MLT: melatonin; NAD(P)H: nicotinamide adenine dinucleotide (phosphate); nSMase2: neutral sphingomyelinase-2; O_2_^−^: superoxide anion; OS: oxidative stress; pO_2_: partial pressure of oxygen; PC: protein carbonyl; ROS: reactive oxygen species; SD: spreading depression; SOD: superoxide dismutase; Tempol: 4-hydroxy-2,2,6,6-tetramethylpiperidine-1-oxyl; TG: trigeminal ganglion; TNF-α: tumor necrosis factor-alpha; TRPA1: transient receptor potential ankyrin type 1; Waves: number of CSD waves. Table note: Directionality reflects whether redox status was reported to trigger CSD (T) or merely shifts its threshold without provoking spontaneous events (M). The interventions were classified into five types (induction, metabolic challenge, pharmacological, microglial targeting, and neurotrophic factors) and compared endogenous changes post-CSD with the effects of different exogenous treatments. SD refers broadly to the phenomenon; here, CSD specifies the cortical variant. Pusic et al. [24] (2015a) and Jiang et al. [15] (2019) both captured bidirectional signaling.*

#### 3.5.1 Acute oxidative wavefronts and MC: context-dependent dynamics

Collectively, the synthesized evidence suggests that CSD generates propagating oxidative wavefronts that operate on a functional spectrum ranging from transient redox signaling to MC depending on the underlying bioenergetic context. High-resolution roGFP imaging of hippocampal slices revealed oxidizing wavefronts that traversed the cornu ammonis area 1 (CA1) stratum pyramidale with variable recovery kinetics. While approximately 40% of the slices demonstrated near-complete redox restoration within the observation window, the remainder exhibited persistent OS, suggesting context-dependent heterogeneity in metabolic resilience [13]. These spatial dynamics were supported by elevated cortical H_2_O_2_ levels and SOD activity in intact animals [30] and were further extended by evidence that oxidative burden is not confined to the cortex but strongly increases the levels of markers in the meninges and trigeminal ganglia [31].

In addition to these associations, under metabolic stress conditions, ROS accumulation may shift from a downstream consequence to a contributing factor. While ROS accumulation is often secondary to depolarization, experimental models of bioenergetic stress have revealed its potential as a causative agent. In the context of chemical glycolytic inhibition (iodoacetamide) or pyruvate substitution, substantial ROS accumulation and abnormal NAD(P)H/FAD oxidation preceded and precipitated MC that culminated in spreading depolarization [14]. The involvement of ROS in this transition was further supported by intracerebroventricular administration of tempol, which strongly reduced the occurrence of CSD [14]. These findings also implicate TRPA1 as a potential signaling node that transduces metabolic stress into altered cortical excitability, particularly when redox signaling is amplified by exogenous oxidative challenge [15].

#### 3.5.2 Biphasic modulation and compartment-specific efficacy

The discordance between the antioxidant dose and CSD kinetics illustrates how compartmentalization may influence treatment effects, with efficacy potentially reflecting localized redox signaling rather than absolute ROS levels. Mechanistic patterns across studies revealed that nonspecific scavenging produces biphasic effects, as clearly exemplified by neonatal melatonin administration [18]. While 10 mg/kg melatonin improved oxidative marker levels and slowed CSD propagation, 40 mg/kg melatonin paradoxically accelerated CSD without altering static MDA or SOD levels [18]. This discordance challenges simplistic, unidirectional ROS reduction models, indicating that indiscriminate scavenging likely disrupts the oxidative eustress required for adaptive ionic signaling.

In contrast, interventions that maintain local redox gradients yield consistent protective effects. Insulin-like growth factor-1 (IGF-1), which acts as an endogenous stabilizer, provides cell type-specific redox buffering by selectively attenuating CSD-induced microglial OS and TNF-α production while preserving astrocytic ROS levels [25]. In hippocampal slice cultures, IGF-1 successfully decreased SD susceptibility while paradoxically increasing spontaneous CA3 bursts [28]. This apparent contradiction, characterized by attenuated susceptibility to SD and increased spontaneous activity, suggests that IGF-1 selectively suppresses the propagating depolarization wave (a pathological event associated with MC) while preserving or even increasing local synaptic transmission. These findings indicate a decoupling of physiological excitability from pathological excitotoxicity [28]. Furthermore, intranasal IGF-1 pretreatment successfully reduced CSD-induced increases in trigeminal MDA levels and CGRP release, reducing overall CSD susceptibility through targeted rather than systemic modulation [29]. This failure of global ROS clearance suggests that CSD vulnerability tracks the cellular source of OS rather than simply its systemic abundance. Consequently, microglia have emerged as the likely cellular compartment driving this vulnerability.

#### 3.5.3 Neuroimmune gating: microglial polarization modulates CSD susceptibility

Across the included studies, microglial polarization consistently emerged as a critical gating mechanism that functionally modulates CSD susceptibility through both redox-dependent and redox-independent mechanisms [23, 24, 26, 27]. Proinflammatory polarization (historically termed M1), modeled through continuous exposure to exogenous interferon-γ (IFN-γ), drives progressive ROS accumulation (as demonstrated by decreased GSH levels), activation of the TNF-α/nSMase2 axis, and disruption of myelin integrity, resulting in a marked 95% reduction in the SD threshold [24].

Furthermore, M2a-like polarization stabilized the cortex against CSD. Basal M2a induction via environmental enrichment (EE) or interleukin-11 (IL-11) improved phenotypic marker expression (with increased arginase-1 (Arg-1)/IL-10 levels and decreased iNOS levels), attenuated ROS-mediated damage, and increased CSD thresholds [23]. Dynamic modulation achieved similar protective effects in the absence of continuous oxidative damage; specifically, stimulation with phasic IFN-γ [27] or dendritic cell-derived exosomes (DC-Exos) [26] successfully reduced iNOS expression and increased CSD thresholds while preserving endogenous GSH levels. Moreover, clodronate-mediated depletion blocked CSD in slice cultures [23], confirming the requirement for microglia without establishing whether this reflects redox signaling or physical interactions. This modal gating mechanism explains why global antioxidants fail in the clinic: they do not recalibrate the metabolic state of microglia, which may remain a chronic driver of cortical hyperexcitability.

### 3.6 Heterogeneity, sensitivity, and subgroup analyses

Biological variability and differences in methodology across animal strains, CSD induction protocols (KCl *vs.* electrical), and redox detection methods precluded meta-analysis. Excluding studies at a high risk of bias [24, 25, 27] and those originating from a single research laboratory [23, 24, 25, 26, 27, 28, 29] did not alter these findings. This robustness persisted in male-only cohorts [13, 14, 15, 18, 29, 30, 31] after P0–10 rat pups were removed [23, 24, 25, 26, 27, 28]. Further subgroup analyses revealed that an increase in ROS levels following CSD challenge was widespread across rat strains, whereas increases in ROS-mediated CSD initiation appeared to occur primarily in murine preparations [14, 15]. Variations in the anesthetic regimen (isoflurane *vs.* urethane) and experimental setup (*in vivo vs. in vitro*) were the primary sources of methodological divergence (Table 1).

## 4. Discussion

Our synthesis suggests that bidirectional CSD–redox interactions occur across time scales (Fig. 4a,b, Ref. [14]). Within minutes, CSD-induced ROS propagate and activate TRPA1/CGRP signaling [15], concurrent with roGFP oxidizing wavefronts traversing CA1 [13]. Acute neuronal bursts recover rapidly under normoxia but persist when metabolic challenge narrows the bioenergetic margin [13]. Over longer time scales, glial remodeling becomes prominent: microglia undergo M2a polarization via EE [23] or phasic IFN-γ stimulation [27], whereas M1 polarization induced by continuous IFN-γ stimulation [24] drives progressive ROS accumulation [25]. High mobility group box 1 (HMGB1) release from neurons occurs as early as after a single CSD episode and predominantly engages astrocytes rather than microglia [33], whereas robust microglial activation is detected only after repeated CSD episodes [33]. This temporal and cellular disconnect suggests that our synthesized evidence captures primarily early-stage compartmentalized neuroimmune priming. Such glial remodeling provides a potential mechanism for interictal hyperexcitability [12], although these findings cannot be validated based on the reviewed evidence.

**Fig. 4.** Mechanisms of reciprocal CSD–redox modulation and compartment-specific interventions. Notes: (a) Proposed feedback loop and metabolic coupling. Illustration of CSD–redox interactions within preclinical models. Inset: High-dose melatonin paradoxically accelerates CSD (U-shaped modulation), disrupting physiological redox tone. Center: Under bioenergetic stress conditions (*e.g.*, glycolytic inhibition), ionic dyshomeostasis and mitochondrial ROS precipitate MC* and sustained depolarization. Right: During the proposed step 2, ROS mediate TRPA1/CGRP activation in meningeal afferents (acknowledging clinical trial limitations), which may trigger IFN-γ-driven M1 polarization (Preclinical Step 3) and amplify CSD susceptibility via TNF-α signaling. (b) Microglial polarization and targeted IGF-1 stabilization. Top: Microglial phenotype modulates CSD thresholds. M1 polarization drives oxidative distress and myelin disruption (MBP↓), lowering thresholds; M2a polarization (EE/phasic IFN-γ) promotes oxidative eustress (GSH↑) and exosomal miR-219 release, increasing thresholds. Bottom: IGF-1 provides compartment-specific stabilization, selectively abrogating microglial ROS/TNF-α signaling while sparing astrocytic signaling pathways to preserve physiological redox gradients. *: MC reflects findings from acute stress models [14] under artificial bioenergetic conditions; TRPA1/CGRP (Step 2) and M1 polarization (Step 3) are proposed preclinical mechanisms with acknowledged translational limitations in human migraine pathophysiology. Ca^2+^: calcium ion; CSD: cortical spreading depression; ROS: reactive oxygen species; MC: metabolic collapse; TRPA1: transient receptor potential ankyrin 1; CGRP: calcitonin gene-related peptide; IFN: interferon; EE: environmental enrichment; IGF: insulin-like growth factor; iNOS: inducible nitric oxide synthase; TNF: tumor necrosis factor; GSH: glutathione; MDA: malondialdehyde; K^+^: potassium ion; Na^+^: sodium ion; IOA: iodoacetate; TVS: trigeminovascular system; TG: trigeminal ganglion; MBP: myelin basic protein.

### 4.1 Compartment-specific stabilization of oxidative eustress

IGF-1 appears to exert stabilizing effects through compartment-specific protective mechanisms, selectively attenuating microglial OS without affecting astrocytic ROS levels [25], increasing CA3 neuronal antioxidant capacity [28], or attenuating trigeminal MDA/CGRP release [29]. These findings underscore that maintaining oxidative eustress (physiological redox signaling for adaptive responses) is more critical than indiscriminate ROS elimination alone. Unlike global scavenging strategies, the compartment-specific approach of IGF-1 appears to preserve these signaling gradients while targeting pathological foci, which represents a distinct mechanism and may explain why broad-spectrum interventions often fail to exhibit clinical efficacy.

### 4.2 Upstream microglial mechanisms and receptor-independent pathways

A systematic review by Anwar *et al*. [34] indicated that SD triggers neuroinflammation but reported insufficient evidence that the inflammatory burden reciprocally alters SD susceptibility, as the included studies demonstrated either no effect or reduced amplitudes. Given that anatomical constraints prevent the direct diffusion of ROS from the cortex to the trigeminal ganglion, we speculate that CSD-derived ROS may be indirectly associated with peripheral nociception via dural neurovascular intermediaries. Specifically, CSD-induced ROS appear to activate TRPA1 in the cortex and meninges [15], thereby triggering local CGRP release and subsequent neurogenic inflammation, which may eventually sensitize trigeminal nociceptive compartments [31]. Such compartment-specific signaling aligns with recent human fMRI evidence showing altered stimulus-induced hemodynamic responses during MA, which has been attributed to neurovascular uncoupling secondary to CSD [35].

We do not propose TRPA1 as a clinical drug target, as systemic antagonists have repeatedly failed in human trials [36, 37]. Paradoxically, this clinical failure may support our framework by revealing that peripheral blockade cannot interrupt centrally driven, compartmentalized redox-metabolic vulnerability. We utilized TRPA1 as a mechanistic node only to understand how ROS transduce metabolic stress into cortical hyperexcitability [15]. This dissociation between preclinical efficacy and clinical failure underscores the necessity of targeting upstream microglial states (*e.g.*, IGF-1-mediated stabilization) over downstream nociceptive receptors. Intranasal IGF-1 exemplifies how stabilizing local cortical redox signaling can prevent downstream headache generation without direct TRPA1 blockade [29], supporting a shift from global scavenging to compartment-specific modulation.

### 4.3 Limitations of global scavenging and compartment-specific alternatives

The observed efficacy mismatch suggests that the clinical failure of broad-spectrum antioxidants reflects compartmental disruption [16, 17] rather than a flawed biological hypothesis. Evidence indicates that nonspecific scavenging disrupts oxidative eustress, the physiological redox baseline essential for neuronal function [9], without resolving oxidative distress within localized cellular compartments. This mismatch is particularly evident in studies in which high-dose melatonin was administered [18], where the observed dissociation between CSD kinetics and the levels of static redox markers suggests the collapse of redox homeostasis. The paradoxical acceleration of CSD despite stable systemic MDA or SOD levels [18] challenges simplistic ROS reduction models, thereby refining our understanding of the redox–CSD framework.

Static markers are insufficient for capturing the compartment-specific dynamics governing CSD kinetics. This uncoupling indicates that indiscriminate scavenging likely disrupts physiological redox homeostasis. Such disruption may involve reductive stress [38, 39], an excessive reductive environment that impairs bioenergetic efficiency. Specifically, high-dose melatonin may trigger receptor desensitization or conditional pro-oxidant effects [40], converting neuroprotection into excitotoxicity. Regardless of the underlying mechanism, therapeutic efficacy depends on stabilizing discrete cellular compartments rather than simply reducing the levels of systemic markers. Without real-time metabolic monitoring, these mechanistic interpretations remain speculative.

In contrast, compartment-specific modulation achieves homeostasis without global ROS elimination. Unlike broad-spectrum scavengers, IFN-γ-DC-Exos reduce iNOS expression without altering TNF-α levels [26], indicating that IFN-γ-DC-Exos selectively, rather than completely, suppress inflammation. Similarly, IGF-1-mediated stabilization and microglial M2a polarization [23, 27] may preserve physiological signaling gradients [9, 41] while preventing MC within pathological foci without disrupting oxidative eustress. This dichotomy explains the reported discrepancies: global clearance abrogates adaptive signaling while pathological foci are left intact, whereas targeted interventions maintain redox homeostasis within the range of oxidative eustress. These findings suggest that microglial phenotype switching and selective mitochondrial protection should be prioritized over global scavengers in the development of future strategies.

### 4.4 Translational feasibility

Our findings have several meaningful clinical implications. First, intranasal IGF-1 attenuates trigeminal CGRP release and the expression of oxidative markers in preclinical models [29], suggesting that a noninvasive delivery route may circumvent the limitations of systemic scavenging strategies. Second, the M2a polarization mechanisms identified here (environmental enrichment paradigms, phasic IFN-γ modulation, and exosomal miR-219 release) [23, 26, 27] are promising in principle, but their pharmacological development for human use remains unrealized. Third, for clinical translation, staged validation is needed, including replication in female rodent models to resolve the 85% male bias [42], integration with noninvasive metabolic monitoring methods (*e.g.*, ^31^P-MRS) [43, 44, 45] to identify patient subgroups with compartment-specific vulnerability, and proof-of-concept trials targeting high-risk windows such as the perimenstrual phases when bioenergetic instability peaks [46, 47].

### 4.5 Sex-specific translational constraints

The evidence presented herein was derived almost exclusively from male rodents (85% of the included studies) [13, 14, 15, 18, 23, 24, 26, 27, 29, 30, 31], which represents a mismatch considering the approximately 3:1 female-to-male prevalence ratio among individuals with clinical migraine [1]. According to the Sex and Gender Equity in Research (SAGER) guidelines [42], this profound sex bias represents a critical translational blind spot that severely limits clinical generalizability. Compared with male brains, female brains have significantly greater resident microglial density, and estrogen suppresses microglial activation. This increased microglial density and hormonal modulation may narrow the functional margin of safety in females, potentially representing a contributing factor to the female-predominant migraine phenotype; however, this mechanism remains untested in females, among whom the disease burden is greater. Furthermore, the estrogen-dependent efficacy of IGF-1 suggested by fundamental neurotrophin interactions remains untested in migraine models. If this efficacy is reproducible in female cohorts, intranasal IGF-1 could serve as a stratified therapeutic strategy during vulnerable windows, such as perimenstrual periods [46, 47], when bioenergetic instability may most substantially compromise CSD threshold resilience, offering an alternative to global ROS scavenging [16, 17].

### 4.6 Methodological considerations and validation

The CSD–redox response was characterized by substantial heterogeneity, arising from the interplay between induction modalities and metabolic states [13, 14, 15, 18, 29, 30, 31]. The reversibility of the redox response differed markedly according to the induction mode. KCl-induced roGFP oxidation partially recovered, whereas hypoxia and the mitochondrial uncoupler carbonyl cyanide-4-(trifluoromethoxy)phenylhydrazone (FCCP) caused persistent oxidation. Ames characterized the brain as operating with a razor-thin bioenergetic “margin of safety” [48]. Malkov *et al*. [14] demonstrated that chemical inhibition of glycolysis converts transient redox shifts into persistent MC. However, because this evidence was derived from a single study using acute slice preparations under extreme artificial bioenergetic stress conditions, generalizing ROS as obligatory triggers of clinical migraine extends beyond the current evidence and requires direct validation.

While seemingly contradictory, methodological variations can be reconciled within a coherent spectrum under a compartmentalization framework. These findings suggest that outcomes depend on specific cellular niches rather than systemic ROS levels, reconciling the distinct efficacies of global and targeted antioxidant strategies. Moreover, the sensitivity of redox measurements depended on the anesthetic regimen used. Isoflurane, which was employed in eight of the thirteen studies (Table 1), substantially reduced susceptibility to CSD compared with urethane, whereas neither anesthetic significantly altered propagation kinetics [49]. These pharmacological discrepancies may have artifactually increased CSD thresholds, potentially masking the potency of pro-oxidant triggers. Furthermore, static oxidative markers (MDA and SOD), despite their inclusion in our four functional parameter groups, failed to capture the transient redox wavefronts that propagate with CSD events.

Additionally, ^31^P-MRS revealed potential interictal phosphorylation deficits in migraineurs [43, 44, 45], which are not consistent with our animal data but are consistent with clinical observations. The chronic bioenergetic instability observed in human patients conceptually aligns with the acute MC reported by Malkov *et al*. [14], suggesting that mitochondrial stabilization may represent translational target. However, the causal link between chronic deficits and acute failure remains untested. Compounding this uncertainty, the 85% male predominance in our synthesized evidence [13, 14, 15, 18, 29, 30, 31] likely obscures sex-specific metabolic vulnerabilities, particularly those shaped by microglial sexual dimorphism [50].

### 4.7 Limitations and future directions

We acknowledge three limitations of this synthesis: the 85% male bias, reliance on acute stress models, and substantial methodological heterogeneity. All the studies had an unclear risk of bias with respect to sequence generation and allocation concealment. Experimental CSD does not replicate spontaneous migraine onset or pain [51]; these models primarily capture aspects of aura neurobiology rather than the complete syndrome.

Future research should transition from static endpoint assays (MDA/SOD) to the real-time dynamic monitoring using probes (roGFP/CellROX) [13, 14]. This shift is essential for capturing the rapid kinetics of oxidative wavefronts. Cell-specific mapping must be implemented to distinguish the distinct contributions of neuronal and glial compartments [23], as tissue-level analyses obscure specific cellular control points. As noted in the Results section, clodronate depletion confirms the necessity of microglia [23] but does not establish whether protection reflects redox signaling or physical interactions, underscoring the need for cell-specific genetic approaches. Furthermore, experimental designs must incorporate female cohorts and adhere to the SAGER guidelines [42]. Researchers must control for ovarian hormone fluctuations, as estrogen modulates both microglial reactivity [52] and CSD susceptibility [21]. Finally, the current evidence primarily establishes correlations. Validating the causal chain requires the integration of optogenetic tools with real-time redox probes to selectively manipulate cell populations while monitoring local redox homeostasis. Such technological convergence is necessary to define the point of no return within the CSD feedback cycle.

## 5. Conclusion

In this systematic review, we identify a bidirectional, context-dependent association between CSD and redox homeostasis in male rodent models. While acute oxidative flux primarily represents a metabolic consequence of CSD, its capacity to reciprocally reduce depolarization thresholds appears to require specific bioenergetic or neuroimmune stress contexts.

The biphasic effects of melatonin demonstrate a disconnect between macroscopic CSD acceleration and static antioxidant capacity, highlighting the functional limitations of nonspecific scavenging strategies. This finding supports the use of IGF-1-mediated compartment-specific protection and microglial M2a polarization as promising alternatives. By reconciling preclinical efficacy with the clinical failure of broad-spectrum scavengers, this synthesis suggests that cell-specific metabolic stabilization should be prioritized over systemic oxidative markers in future trials.

The critical limitations of the current evidence include the predominance of male subjects (85% of the study population) and the reliance of a single study on MC. These findings highlight three priorities for future research: (ⅰ) reconsidering the reliance on systemic markers alone, (ⅱ) prioritizing microglial M2a modulators, and (ⅲ) developing compartment-specific stabilizers (*e.g.*, intranasal IGF-1). If these findings are validated in female cohorts and through sex-specific profiling, the targeted modulation of this upstream redox–immune axis may represent a promising strategy for addressing MA. Immediate translational priorities include validating the use of intranasal IGF-1 in female migraine models and developing noninvasive metabolic monitoring methods to identify patient subgroups with compartment-specific vulnerability, ultimately reinforcing the idea that successful interventions involve engaging context-dependent mechanisms rather than pursuing universal ROS elimination.

## Supplementary Material

Supplementary material associated with this article can be found, in the online version, at https://files.jofph.com/files/article/2098294025658220544/attachment/Supplementary%20material.zip.








