# Supplementary material for: Compartment-specific redox modulation in migraine with aura: a systematic review of context-dependent mechanisms
Source: J Oral Facial Pain Headache. 2026 Sep 12;40(5):39–53. doi: 10.22514/jofph.2026.059 (PMC13678554; doi:10.22514/jofph.2026.059)
Supplement: Supplementary file 2 [file Supplementary-material-3.pdf]

Study Reference	Initial Sample Size	Animal Source	Housing Conditions
Viggiano,A et al.(2011)	Total: 15 rats<br>Group 1: 5 rats (O_2^- evaluation)<br>Group 2: 5 rats (H_2O_2 evaluation)<br>Group 3: 5 rats (SOD activity)	Not specified	• Temperature: 22±1°C;• Humidity: 70%;• Light-dark cycle: 12h/12h (7:00-19:00);• Caging: Paired housing;• Enrichment: Not specified
Grinberg,Y.Y et al.(2012)	Not specified	Charles River, Wilmington, MA, USA	Slice Culture Conditions:-Media: Serum-free neurobasal-based formula (detailed composition provided)；Incubation: Standard lab conditions (37°C, 5% CO_2)；Maintenance:Electrophysiological validation of slice viability
Grinberg,Y.Y et al.(2013)	Total Live Pups: ~120 (12 dams × 10 pups/litter)<br>Experimental Unit (Slice Cultures): Varies per experiment (e.g., Fig 4c: Control n=37slices, TNFα n=10slices)	Charles River (Wilmington, MA, USA)	• Temp/Humidity/Light Cycle/Pup Housing: Not specified；• Cage Type (Dams): Single-housed with Enviro-dri® paper bedding & Nestlets
Shatillo,A et al.(2013)	CSD experiments: Total animals: 11<br>Groups: CSD group (n=6), Sham control (n=5)<br>Electrophysiology: n=4–6 hemiskull preparations	CSD experiments: National Laboratory Animal Center, University of Eastern Finland	Compliance with EU Directive 86/609/EEC (temperature/humidity/light cycle details Not specified)
Malkov,A et al.(2014)	In vitro: 40 slices (from multiple mice)<br>In vivo: 5 mice	Not specified	Surgical: Isoflurane anesthesia<br>In vivo: Heating pad (37°C)
Pusic,K.M et al.(2014)	Initial: EE= 12, NE= 12	Charles River Laboratories	EE: Group-housed in Marlau-style cages with running wheels and dynamic mazes (changed 3x/week)<br>NE: Single-housed standard conditions<br>Temperature/Humidity/Light Cycle: Not specified
Pusic,A.D et al.(2015a)	Pregnant females: 12 rats (10 pups/litter);<br>In vivo groups: n = 3–4 animals/group	Charles River Laboratories	Pregnant rats: Single-housed with Enviro-dri paper bedding & Nestlets;<br>Postnatal: Standard lab conditions (details Not specified)
Pusic,A.D et al.(2015b)	- In vitro (slices): n=3–17/group (e.g., Fig 1F control n=17)<br>- In vivo: n=2–6/group (e.g., Fig 5D n=2–3)	Charles River Laboratories	- In vivo: Not specified (only prenatal dams described)<br>- In vitro slices: 36°C, 95% humidity, 5% CO_2<br>- Bedding: Enviro-dri + Nestlets (for dams)
Pusic,K.M et al.(2019)	1. Hippocampal slices: n=8/group<br>2. In vivo SD: Sham n=3, Unstim n=3, IFNγ n=5<br>3. Protein carbonyl: Sham n=7, IFNγ n=5	Charles River Laboratories	1. Standard housing (unspecified temp/humidity/light cycle);2. Bedding: Enviro-dri paper;3. Nesting: Nestlets;4. Adults: Double-housed；5. Surgical: 37°C + 95-100% O_2
Jiang, L et al.(2019)	Rats: 30;Mice: 36<br>Group Details (Based on Experimental Design): In Vivo Rat Study (i.c.v.perfusion & CSD): Anti-TRPA1 Ab group: n=10;Anti-IgG Ab (CSD control): n=8;Anti-IgG Ab (Sham control): n=7<br>IHC: Rats: n=3;Mice: n=3 In Vitro Mouse Study (Brain Slice): Series 1: Multiple groups (n=6-8 per group) ;Series 2: Multiple groups (n=6 per group)	Shanghai SLAC Laboratory Animal Corporation Ltd.	Temperature, humidity, light cycle, cage type, enrichment measures Not specified in the text.
Won,L et al.(2020)	Total n=90; Biological replicates: 5-8/group (Subject to the detection indicators)	Charles River Laboratories	• Static micro isolator cages;• Corn cob bedding;• Enviro-dri + nestlets;• 12h light-dark cycle ;• Controlled humidity/temperature
Araújo,A.D.O et al.(2023)	Total: 40 rats<br>Per Group: 10 rats (4 groups: Intact, Vehicle, MLT-10, MLT-40)	Sigma Aldrich	Temperature: 23°C ± 1°C<br>Light Cycle: 12-h light/dark (lights on at 7 PM)<br>Cage Type: Polypropylene (51 × 35.5 × 18.5 cm)<br>Enrichment: Standard bedding; no specific enrichment mentioned
Ackermann,M.A et al.(2024)	Total: 65 mice (typically 3–4 slices per brain)	Bred and maintained at the central animal facility of University Medical Center Göttingen	- Regular diet: V1124-0 (SSNIFF)<br>- AO-enriched diet: 250 mg/kg α-lipoic acid, 2.5 g/kg NAC, 125 mg/kg vitamin E

Study Reference	Group Design	Final Sample Size	Intervention	Control Groups	Time Points	Ethical Approval （YES/POR）
Viggiano,A et al.(2011)	Group 1: O_2 evaluation via DHE fluorescence (n=5);Group 2: H_2O_2 evaluation via microdialysis/HPLC (n=5);Group 3: SOD activity via histochemistry (n=5)	n=5 per group (Total N=15 rats)	Type: Chemical CSD induction<br>Dose: 3M KCl (saturated filter paper)<br>Duration: 20-min continuous application<br>Route: Topical on exposed cortex	Type: Internal control (contralateral hemisphere)<br>Matching: Same animal, homologous cortical region	Baseline: 2h pre-intervention stabilization<br>Post-CSD: O_2/SOD at 1h post-KCl; H_2O_2 at 0-15/15-30/30-45/45-60 min intervals	Yes（Ethical approval number：Not mentioned）
Grinberg,Y.Y et al.(2012)	Control, Acute IGF-1 (15-30 min), 3-day IGF-1, 7-day IGF-1, Insulin (400 μg/mL), Ascorbate (2 mM), H_2O_2(50/200 μM), Menadione (50 μmol/L), IGF-1 + Ascorbate/H_2O_2co-treatment	Fig1d: Control n=6, Acute IGF-1 n=7; Fig1e: Control n=8, 3-day IGF-1 n=7; Fig3c: Control n=14, IGF-1 n=16, IGF-1+50μM H_2O_2 n=5, IGF-1+200μM H_2O_2 n=9; Statistical range: n=3-18/group	- Type: IGF-1 (40 ng/mL or 100 ng/mL), Insulin (400 μg/mL), Ascorbate (2 mM), H_2O_2 (50/200 μM), Menadione (50 μmol/L)<br>- Duration: Acute (15-30 min), 3 days, 7 days (phasic: day IGF-1/night normal media)<br>- Route: Serum-free media supplementation	Sham controls: "sham control cultures only experienced normal media" (matched by same-day culture)	- SD induction: 6 times/hour for OS experiments<br>- OS measurement: 24h post-SD<br>- Bursting recording: During baseline (unstimulated)	Yes（Ethical approval number：Not mentioned）
Grinberg,Y.Y et al.(2013)	1. Cell-specific OS: Sham vs SD (Fig2);2. IGF-1 effect: SD vs SD+IGF-1 (Fig3);3. TNF-α effect: Control vs TNF-α vs TNF-α +IGF-1 (Fig4b);4. SD threshold: Baseline vs Post-SD ± sTNFR1 (Fig4d,e)	- Fig2 (OS): n=3-4 slices/group ;- Fig4a (TNF-α): n=3-4 slices; - Fig4c (Threshold): Control n=37 (pooled), TNF-α n=10; C5- Fig4d (Post-SD): n=13-17	- IGF-1: 100 ng/mL (≈13.2 nM), 3d pretreatment, media<br>- TNF-α: 100 ng/mL, 24h (OS) or 3d (threshold), media<br>- sTNFR1: 200 ng/mL, 3d post-SD, media	1. Sham: Half-maximal stimulation<br>2. Untreated: No additives<br>3. Fixative control: No LPS (Fig1c)<br>4. Antibody/isolectin control: Omit primary antibody or isolectin	- OS/TNF-α: 24h/6h post-SD<br>- SD threshold: Baseline & 3d post-SD<br>- Microglial movement: 24h post-SD	Yes（Ethical approval number：Not mentioned）
Shatillo,A et al.(2013)	Experimental group: KCl-induced CSD (n=6);Control group: Sham surgery + saline application (n=5)	- CSD group: n=6 rats (LFP/MDA);- Sham control: n=5 rats;- Ex vivo hemiskull: n=4–6 preparations;- CGRP assay: Not mentioned (cultured DRG neurons)	Intervention - Agent: 1M KCl<br>- Volume: 5μl<br>- Timing: After 5-min baseline recording<br>- Site: Dura mater via cranial window	- Type: Sham + saline<br>- Matching: Identical surgery and sampling procedures	- Tissue sampling: Post-CSD (after 1-h recording)<br>- H_2O_2 stimulation: Peak effect at 12 min<br>- CGRP assay: 15-min exposure	Yes（Ethical approval number：Not mentioned）
Malkov,A et al.(2014)	1. Glucose-ACSF (control);2. Pyruvate-ACSF;3. Pyruvate-ACSF + IOA;4. Pyruvate-ACSF + Tempol;5. In vivo KCl (baseline) ;6. In vivo KCl + Tempol	- In vitro:• Glucose-ACSF: 43 slices; • Pyruvate-ACSF: 40 slices (14/40 showed MC);• Pyruvate+IOA: 17 slices (9 elec. stim. + 8 spont.);• Pyruvate+Tempol: 11 slices<br>- In vivo: 5 mice (self-controlled design)	- Tempol:<br>• In vitro: 2 mM perfusion<br>• In vivo: 1 M (0.4 μL ICV + topical 5 μL under KCl cotton)<br>- IOA: Glycolysis inhibitor (conc. NR)<br>- Stimulation: 100 Hz/1s (electrical) or 10 Hz/5s (glutamate puff)	- Glucose-ACSF (energy substrate control)<br>- Pre-intervention baseline (in vivo self-control)	- In vitro: Pre-stim baseline → Stimulation (sec) → Recovery (20-30 min)<br>- in vivo:CSD counts over 1 hr	Yes（No.30-03102012）
Pusic,K.M et al.(2014)	1. EE group: 12 male Wistar rats group-housed in Marlau-style enriched cages (running wheel + changed maze 3x/week) for 35 days;2. NE group: 12 age-matched rats single-housed under standard conditions;3. Intranasal IL-11 group: 1mg recombinant rat IL-11;4. Sham group: Sodium succinate buffer	- EE/NE electrophysiology: n=5-6;- EE/NE biochemistry: n=3;- Slice culture microglia ablation: n=6-12/group;- In vitro IL-11: n=8;- Intranasal IL-11: n=5 (electrophysiology), n=3 (biochemistry)<br>microglia quantification n=9/group	- EE: Multilayer cage (58×88×65cm), running wheel + 6 maze variations changed 3x/week × 35d<br>- Drugs: In vitro IL-11 (100ng/mL × 3d); Minocycline (10μg/mL acute); Intranasal IL-11 (1mg/50μL over 20min via alternating nostrils)	- NE: Standard single housing<br>- IL-11 Sham: Sodium succinate buffer<br>- In vitro: Untreated + vehicle controls （Not mentioned）	- Post-EE: 35 days<br>- Post-intranasal IL-11: 24hr<br>- In vitro treatments: IL-11 (3d), Minocycline (acute)	Yes（Ethical approval number：Not mentioned）
Pusic,A.D et al.(2015a)	In vitro: Hippocampal slice cultures from P9-P10 Wistar rat pups (either sex), maintained for 21–35 days in vitro (DIV). In vivo: Male Wistar rats (300–400 g). SD induced via electrical stimulation (slices: 1000 μA current) or KCl microinjection (neocortex). Slice vitality ≥95% (Sytox assay).	- Fig 1B: Control (n=14), SD 1d (n=5), SD 3d (n=4), SD 7d (n=18).; - Fig 1E: Control (n=3 slices), SD 1d (n=3), SD 7d (n=3).;- Fig 2C: Control (n=5), IFNγ (n=5).;- Fig 2K: Control (n=12), IFNγ (n=9).; - Fig 3C: Control (n=9), GW4869 (n=8), GW4869+SD (n=3), GW4869+IFNγ (n=5).; - Fig 4D/E/N/Q: n=3–4 animals/group.	- SD in slices: 6 SDs via 1000 μA current (10 pulses, 10 Hz) every 7–9 min over 1 h.<br>- IFNγ: 500 U/mL × 12–24 h (non-toxic per Sytox).<br>- Anti-CD4: 10 μg/mL × 3 days (clone OX-38).<br>- GW4869: 10 μM × 1 h pre-treatment + maintained.<br>- In vivo SD: 6 neocortical SDs via KCl.	- Slices: Age-matched cultures with sham recording (evoked field potentials);- In vivo: Contralateral neocortex (no SD);- Antibody control: Mouse IgG2a isotype (#MG2a00;).	- Acute: OS (3–7 h post-IFNγ).<br>- Short-term: MBP loss (1d post-SD/IFNγ).<br>- Recovery: MBP (3d/7d post-SD).<br>- In vivo: All endpoints at 1d post-SD.	Yes（Ethical approval number：Not mentioned）
Pusic,A.D et al.(2015b)	In vitro: (1) Untreated control; (2) Phasic IFNγ (500 U/mL, 12h/day ×7d); (3) Pulsed IFNγ (single 12h IFNγ); (4) Exosomes from IFNγ-stimulated slice cultures; (5) Exosomes from IFNγ-stimulated microglia.;In vivo: (6) Sham (nasal buffer); (7) Nasal IFNγ (50,000 U single dose).	Representative examples:- Phasic IFNγ MBP: n=6 (Fig 1A); - Pulsed IFNγ OS: n=11–17 (Fig 1F); - Exosome-treated SDT: n=8 (Fig 2D); - Nasal IFNγ FluoroMyelin: n=3–6 (Fig 5A);Note: n varies per experiment; values from figure legends.	Type: Recombinant rat IFNγ (R&D Systems) or exosomes.<br>Dose: 500 U/mL (in vitro); 50,000 U (nasal, scaled from in vitro).<br>Time: 12h pulses (in vitro); single dose (nasal).<br>Route: Culture media (in vitro); nasal instillation (in vivo).	In vitro: Age-matched untreated slice cultures.;In vivo: Nasal sodium succinate buffer;Matching: Identical age (e.g., slices at 21–35 DIV) and handling procedures.	In vitro: 3d & 7d post-IFNγ; 7d post-exosomes.<br>In vivo: 1d & 3d post-nasal IFNγ.<br>Rationale: Maximal effects at 7d (stated in Results).	Yes（Ethical approval number：Not mentioned）
Pusic,K.M et al.(2019)	In vitro:;- Control (Untreated slices, n=8);- IFNγ-DC-Exos (100 μg, n=8)<br>In vivo (SD threshold):- Sham (Sodium succinate vehicle, n=3–5);- Unstim-DC-Exos (n=3–5);- IFNγ-DC-Exos (100 μg, n=3–5)<br>In vivo (Oxidative stress):- Sham (Vehicle, n=7);- IFNγ-DC-Exos (n=5)	- Slice cultures: Control (n=8), IFNγ-DC-Exos (n=8);- In vivo SD: Sham (n=3–5), Unstim-DC-Exos (n=3–5), IFNγ-DC-Exos (n=3–5);- Protein carbonyl: Sham (n=7), IFNγ-DC-Exos (n=5)	Type: IFNγ-stimulated dendritic cell exosomes (IFNγ-DC-Exos);Dose: 100 μg<br>Time:- Slice cultures: 3 days incubation;- In vivo: Single dose, measured 1 day (SD) or 3 days (ROS) post-administration<br>Route:- Slice cultures: Added to culture media- In vivo: Intranasal (5 μL/2 min per nostril, total 50 μL over 20 min)	Types:- Negative control: Untreated slices/rats or vehicle (sodium succinate);- Activity control: Unstim-DC-Exos (in vivo SD)<br>Matching:- All controls age-matched;- Slice cultures: Matched DIV (21–35 days)	- SD threshold:<br>- Slice cultures: 3 days post-treatment<br>- In vivo: 1 day post-administration<br>- Oxidative stress: 3 days post-administration<br>- Gene expression: 1 day post-administration	Yes（Ethical approval number：Not mentioned）
Jiang, L et al.(2019)	·In vivo (rat):- Anti-TRPA1 Ab (i, n=10);- Anti-IgG Ab (ii, n=8);- Sham (iii, n=7)<br>·In vitro (mouse slice):- Series 1:• Krebs (i, n=6);• Tempol 3mM (ii, n=7);• H_2O_2 50μM (iii, n=7);• Tempol + H_2O_2 (iv, n=7);• Tempol + UMB 15μM (v, n=6);• H_2O_2 + A967079 1μM (vi, n=6);- Series 2:• Anti-IgG Ab 0.025μM (xiii, n=6);• Anti-CGRP Ab 0.4μM (xii, n=6);• Anti-CGRP Ab + AITC 50μM (xi, n=6);• Anti-CGRP Ab + H_2O_2 50μM (x, n=6)	Rat:- Anti-TRPA1 Ab: n=10;- Anti-IgG Ab: n=8<br>- Sham: n=7;Mouse slice:- Series 1: n=6-7/group;- Series 2: n=6/group;Contradiction：Total rats=29 ≠ Group sum=25 (10+8+7)	Type: Antibodies (anti-TRPA1, anti-CGRP), drugs (Tempol, H_2O_2, UMB, A967079, AITC)<br>Dose:- Anti-TRPA1 Ab: 0.8μg total;- H_2O_2: 50μM<br>Time:- Antibody: 4 days pre-CSD (rat);- Drugs: 30-45 min pre-incubation (mouse)<br>Route:- i.c.v. perfusion (rat);- Slice perfusion (mouse)	Type:- Active control: Anti-IgG Ab;- Sham: No KCl induction;Matching:- Same antibody dose (0.8μg);- Identical surgical procedures	- MDA: Immediately after CSD induction<br>- CSD: During 30-min KCl induction (rat) / Post-drug incubation (mouse slice)	Yes（Ethical approval number：Not mentioned）
Won,L et al.(2020)	1. Recurrent SD + IGF-1 pretreatment<br>2. Recurrent SD + Vehicle (succinate buffer) pretreatment<br>3. Sham (0.5M NaCl injection) + Vehicle<br>4. Naive animals (no SD) for baseline CGRP measurement	TG MDA/CGRP: n=5/group (Fig 1E,H)<br>; TCC c-fos: n=5-7/group (Fig 1K); Blood glucose: n=8/group (0h), n=4/group (other times); Naive CGRP: n=7/group (Fig 5)	Type: Human recombinant IGF-1 (R&D Systems #191-G1)<br>Dose: 150 μg<br>Time: 24 hrs before SD induction<br>Route: Intranasal (50μl total, 5μl/alternating nostril every 2 min under isoflurane anesthesia)	1. Vehicle control: 50μl succinate buffer (identical volume/route);2. Sham control: 0.5M NaCl microinjection instead of KCl;3. Procedure control: Naive animals without surgery/SD	- Blood glucose: 0h (pre-IGF-1), 0.3h, 1h, 3h, 6h, 24h<br>- Tissue harvest: After 90-min SD protocol (immediately post-SD)	Yes（Ethical approval number：Not mentioned）
Araújo,A.D.O et al.(2023)	- Intact group: Not mentioned<br>- Vehicle group: Saline + 5% ethanol solution<br>- MLT-10 group: 10 mg/kg melatonin<br>- MLT-40 group: 40 mg/kg melatonin	n = 10 per group (total 40 male rats)	Type: Melatonin (Sigma Aldrich);Dose: 10 mg/kg or 40 mg/kg;Time: Every other day from PND7–27 (11 administrations total);Route: Intraperitoneal injection<br>Vehicle: 0.1 mL saline + 5% ethanol	Type: Dual controls (untreated + vehicle)<br>Matching: Same age, sex, litter origin (pups from 3–5 distinct litters per group)	Body weight: PND7,14,21,28<br>Glycemia: PND27 (4h pre- & post-MLT)<br>Behavior: PND30 (OF-anxiety), PND31 (position memory), PND32 (shape memory), PND34 (EPM)<br>CSD: PND36–42<br>MDA/SOD: After CSD recording on PND36–42	Yes(approval protocol no. 0006/2020)
Ackermann,M.A et al.(2024)	- Normoxic SD: K^+ microinjection (3M KCl)<br>- Hypoxic SD (HSD): O_2 withdrawal + 2mM Na_2SO_3<br>- Chemically-induced SD: FCCP (2μM mitochondrial uncoupler)<br>- Subgroups: Ca^{2+}-free ACSF, DPI (20μM), allopurinol (200μ M), AO-enriched diet	Total mice: 65 (explicitly stated)	- K^+-SD: 3M KCl microinjection (1.5s, 0.25bar, stratum radiatum);- HSD: ACSF saturated with 95% N_2/5% CO_2 + 2mM Na_2SO_3;- FCCP-SD: 2μM in ACSF;- Drug pretreatment: 15min (e.g., DPI)	- Wild-type slices: Autofluorescence control (Fig 1H);- Solvent control: 0.2% DMSO (Fig 1G);- Baseline control: Pre-treatment period for each slice	- Baseline: ≥90min post-slice recovery<br>- SD quantification: At ΔV_o nadir or 300s post-onset (if no peak)<br>- Long-term: fEPSP recorded every 30s	Yes（Ethical approval number：Not mentioned）

Study Reference	CSD Induction Method + Site	Inducer Parameters	Induction Timepoint	Anesthesia (Type + Monitoring)	Model Success Rate + Exclusion Criteria	Model Validation
Viggiano,A et al.(2011)	Chemical induction by continuous application of 3M KCl-soaked paper on cortical surface (0 mm anterior to lambda, 4 mm lateral to midline)	3M KCl solution (confirmed no contralateral CSD induction);20-min continuous application on the exposed cortical surface	20 min continuous application	Pentobarbital (50 mg/kg, i.p.)；Depth monitored by stable EEG/DC potential recording	Success: ≥6±2 CSD waves in treated cortex；Exclusion: Absence of CSD waves on EEG/DC recording	• EEG amplitude decrease + slow DC depolarization waves (8±2 mV amplitude, 2.3±0.4 min duration)<br>• Contralateral cortex shows stable DC and normal EEG
Grinberg,Y.Y et al.(2012)	Electrical stimulation of dentate gyrus to induce SD in CA3 region of rat hippocampal slice cultures.	• Stimulus: 10 pulses at 10 Hz (100 μ s/pulse) ;• Current: Incrementally increased (start: half-maximal field potential current; max: 10,000 nC)<br>•Current adjustment interval:1-2 minutes(current doubled and re-applied if	SD induced after: • Acute agent exposure (15-30 min pre-test) ;• 3-day/7-day pretreatment;• 30 min post-oxidant exposure (e.g., H_2O_2)	Not mentioned (in vitro model)	Inclusion: CA3 field postsynaptic potential ≥3	• SD confirmed by depolarization waves (Fig 1b-c)<br>• OS quantified via CellROX™ in CA3 (Fig 2)<br>• Hyperexcitability graded: 1(normal)-3(SD) (Fig 4a)
Grinberg,Y.Y et al.(2013)	Electrical stimulation at dentate gyrus; recording at CA3 pyramidal layer.	Inducer Parameters • Pulses: 10 × 100 μ s pulses, frequency 10 Hz • Intensity: Initial current = half-maximal field potential-eliciting current, doubled gradually (reapplied after 1–2 min if SD	Experiments at 21–35 days in vitro (post-culture) ; Total SD induction duration:6 SDs completed within 1 hour	N/A (In vitro model)	Slices discarded if field potentials <3 mV (10–20 μA stimuli)	1. ↑ OS (CellROX™/protein carbonyls; Fig. 1d)<br>2. ↑ TNF-α (Fig. 4a)<br>3. ↓ SD threshold post-SD (Fig. 4d)
Shatillo,A et al.(2013)	Local application of 1 M KCl (5 μl) through cranial window to intact dura mater over right visual cortex.	1 M KCl solution, volume: 5 μl.	Applied after 5 min of baseline recording (LFP/fMRI).	Surgery: Isoflurane (4% induction, 2% maintenance)<br>Recording: Urethane (1.25 g/kg i.p.) administered after 15-min recovery from isoflurane (to avoid cortical/subcortical function interference)	Success: 100% (6/6 KCl-treated rats showed CSD);Exclusion: Sham controls (saline, n=5) excluded from CSD group analysis.	Imaging: BOLD fMRI mapped CSD propagation toward trigeminal ganglia (Fig 1D)<br>Electrophysiology: LFP quantified wave features (amplitude 31.2±6.2 mV, duration 46.7±6.4 s); H_2O_2-induced trigeminal nerve firing (blocked by TRPA1 antagonist TCS-5861528)<br>Biochemical: ① Ipsilateral cortex/meninges & bilateral
Malkov,A et al.(2014)	Focal topical application of 3 mol/L KCl via cotton ball on pial surface	Parieto-occipital cortex (stereotaxic coordinates: posterior 3.8 mm, lateral 2 mm from bregma)	• KCl concentration: 3 mol/L;• Application volume: < 50 μL per cotton ball;• Replacement interval: Every 30 min	After surgical stabilization (craniotomy + electrode implantation + saline coverage)	• Type: Ketamine/Xylazine cocktail (Ket-Xyl-PB = 6:1:20, 10 mL/kg i.p.)• Depth monitoring: Not mentioned	100% acute induction (5/5 mice exhibited recurrent CSD)
Pusic,K.M et al.(2014)	• In vitro (hippocampal slices):<br>- Trans-synaptic induction: Bipolar stimulating electrode at dentate gyrus, DC recording electrode in CA3 stratum pyramidale<br>- Chemical induction: KCl-agar wand (1M) applied to dentate gyrus<br>• In vivo (whole animal):<br>- KCl microinjection into neocortex via craniotomy (-2.0 mm posterior to Bregma, 1.5 mm lateral to sagittal suture)	• Electrical stimulation:<br>- 10 pulses at 10 Hz (100 μs/pulse)<br>- Starting current: 50% of maximal field potential-eliciting current; - Incremental increase per trial (3-min intervals)• KCl application (in vitro):- 1M KCl in 3.5% agar; - 1-2 sec contact duration;• KCl application (in vivo): - 0.5M KCl solution; - Pressure injection (Picospritzer-II system); - Volume calibrated via oil displacement measurement;-Injection duration doubled every 4-5 min until SDtriggered	• In vitro treatments (minocycline/IL-11/conditioned media): 3 days post-treatment (to allow protein synthesis-dependent effects);• Environmental enrichment (EE): After 35 days of EE exposure;• Intranasal IL-11 administration: 24 hours post-administration (to match peak effect)	Slice preparation: Progressive anesthesia with 100% CO_2 followed by decapitation;• In vivo surgery/recording:<br>- Isoflurane (5% induction, 3% during surgery, 2-3% during recording)<br>- volitional breathing<br>- Depth confirmation: Absence of paw pinch withdrawal reflex; - Continuous arterial oxygen monitoring (95-100% saturation)	• Viability standard: Sytox staining exclusion of cultures with dead neurons in stratum pyramidale• Inclusion criteria: Field EPSP amplitude >3 mV in CA3 region<br>• Exclusion criteria: - Cultures treated with penicillin/streptomycin (induces seizures) ;- Oxygen concentration >20% in recording chamber (induces seizures); - Cultures showing bursting/seizure activity upon stimulation	• SD confirmation: Characteristic DC potential shift with concomitant suppression of spontaneous neuronal activity:• Microglia ablation: - >95% reduction in Iba1+ cells (immunohistochemistry): - No change in NeuN/GFAP/CNPase staining;- SD susceptibility restored by adding exogenous microglia (rescue experiment);• Polarization state:- Immunohistochemistry (CD32 for M1, Arg-1 for M2a); - RT-qPCR with >2-fold change threshold for significance (e.g., iNOS↓, TGFβ ↑ , IL-10↑)
Pusic,A.D et al.(2015a)	In Vitro (Hippocampal Slice Cultures)<br>• Electrical stimulation: 10 pulses/10 Hz (100 μs/pulse)<br>• Site: CA3 pyramidal neuron interstitial area (genu of the CA3 interstitial pyramidal neuron area)<br>In Vivo (Neocortex)<br>• Chemical induction: Pressure microinjection of KCl (0.5 M)<br>• Site: Neocortex (6.0 mm caudal to Bregma, 4.5 mm lateral to sagittal suture, 750 μm below pial surface)	In Vitro (Hippocampal Slice Cultures)<br>• Current: 1000 μA (threshold-calibrated)<br>• Pulse width: 100 μs<br>In Vivo (Neocortex)<br>• KCl volume: 2–3× baseline volume (baseline calculated via oil-displacement microscopy; value Not mentioned)	In Vitro (Hippocampal Slice Cultures)<br>• Cultures aged 21–35 days (in vitro)<br>In Vivo (Neocortex)<br>• Adult male Wistar rats (300–400 g)	In Vitro (Hippocampal Slice Cultures)<br>• Not mentioned (ex vivo tissue)<br>In Vivo (Neocortex);• Isoflurane concentrations:- Induction: 5%;- Surgery: 3%;- Recording: 2–3%;• Core temperature maintenance: 37°C ("kept warm with an overhead infrared lamp")<br>• Depth monitoring: No physiological metrics(e.g., respiratory rate, reflexes) beyond gas concentration control.	In Vitro (Hippocampal Slice Cultures)：<br>Inclusion: CA3 field postsynaptic potential ≥3 mV<br>(5–20 μA stimulation) + slice vitality ≥95%<br>(Sytox staining);Method described as "highly reproducible"; No additional explicit exclusion criteria<br>In Vivo (Neocortex)：Not mentioned (no success rate criteria or quantitative exclusion data provided in text)	Electrophysiology:• DC potential shift (Fig 1A)<br>• 6 SDs induced within 1 hour;Structural/Molecular:<br>• MBP loss (Western blot, Fig 1B);• Myelin ultrastructural disruption (EM, Fig 1D);Oxidative stress (CellROX) + glutathione level (ThiolTracker) detection (in vitro);In Vivo (Neocortex);Electrophysiology:• DC potential shift (Fig 4A);Functional/Cellular:• Increased nSMase activity (Fig 4E);• T-cell infiltration (Fig 4N);• Reduced MBP immunostaining (Fig 4G,K) Protein carbonylation quantification (OS readout)
Pusic,A.D et al.(2015b)	- In vitro: Bipolar electrical stimulation at dentate gyrus (hippocampal slice cultures)<br>- In vivo: Focal KCl microinjection in neocortex (750 μm below pial surface)	- Electrical: Stimulus protocol: 10 pulses at 10 Hz (100 μs/pulse), initial current based on half-maximal field potential, max 10,000 nC<br>- Chemical: 0.5 M KCl, pressure injection via Picospritzer-II, volume calibrated post-hoc in oil	- In vitro: 3 or 7 days post-treatment (7 days for maximal effect)<br>- In vivo: SDT measured at 1 day post-nasal IFNγ (*myelin markers at 1/3 days)	- In vivo: Isoflurane (5% induction, 1.5–3% maintenance in oxygen), core temperature 37°C (thermoregulator + IR lamp), no depth monitoring device described	Inclusion Criteria (in vitro): CA3 field<br>postsynaptic potential ≥3 mV (5–20 μA<br>stimulation) + slice vitality >95% (SYTOX staining)<br>Exclusion Criteria (in vitro): Slices with >5% dead cells (SYTOX staining)<br>SD Failure Definition: Threshold set to 10,000 nC if no SD evoked	Primary: Transient negative DC shift recorded by 2–4 μ m tip microelectrode (CA3/in vivo neocortex)<br>Secondary:<br>Myelin markers: MBP (Western blot, in vitro) + FluoroMyelin staining (in vivo)<br>Oxidative stress: Reduced protein carbonyls (Abcam kit #ab12687, in vivo) + CellROX Deep Red detection (in vitro
Pusic,K.M et al.(2019)	In vitro: Hippocampal slice cultures - Bipolar electrical stimulation electrode positioned in slice.DC recording in CA3 stratum pyramidale.<br>In vivo: Rats - KCl microinjection at anterior craniotomy site: -2.0 mm from bregma, 1.5 mm left of sagittal suture. Recording at posterior site: -6.0 mm from bregma, 4.5 mm lateral,750 μm below pial surface.	In vitro: Electrical Stimulus: 10 pulses, 10 Hz, 100 μs/pulse. Intensity started at half-maximal field potential and increased stepwise.<br>In vivo: 0.5 M KCl solution. Pressure ejection via Picospritzer-II. Injection duration doubled every 4-5 min until SD triggered. Volume estimated by injection into oil.	In vitro (Slice Treatment): SD threshold measured 3 days after exosome application to culture media<br>In vivo (Rat Treatment): SD threshold measured 1 day after intranasal administration of exosomes/vehicle.	In vivo recordings: Isoflurane in O_2 (5% induction; 2-3% maintenance during recording). Depth monitored by: 1. Pulse oximetry (SpO_2 95-100%);2. Respiratory rate/depth;3. Animal color;4. Absence of withdrawal to hind paw pinch	Slice Cultures: Excluded if any Sytox-positive (dead) pyramidal cells observed. Specific induction success rate Not mentioned.<br>In vivo: No explicit exclusion criteria or success rate provided for SD induction. All reported data are from successfully induced SD events.	1. Theoretical Basis: SD established as model for migraine aura/headache (Introduction, Page 1).<br>2. Consistency: Low, stable SD threshold in controls (Sham: 1.00 ± 0.14; Fig 1B).3. Responsiveness: IFNγ-DC-Exos induced >12-fold (in vitro) and 57-fold (in vivo)threshold increase (Fig 1A, B).4. Mechanistic Plausibility: Reduced oxidative stress (iNOS↓, carbonyls↓; Fig 2) aligns with known SD pathophysiology.
Jiang, L et al.(2019)	In vivo (rat):<br>• K^+ application on parietal cerebral cortex<br>• Coordinates: 5 mm posterior and 2 mm lateral to bregma with dura intact<br>In vitro (mouse):<br>• KCl ejection in somatosensory region of coronal brain slices	In vivo:• 2 M KCl, continuous perfusion for 30 min<br>In vitro:• Submaximal CSD: 50 mM KCl (Series 1);• Full CSD: 260 mM KCl (Series 2)	In vivo:• Day 4 after antibody i.c.v. perfusion<br>In vitro:• Submaximal CSD: 45-min interval between episodes;• Full CSD: 2-h interval between episodes	In vivo:• Isoflurane (5% in O_2:N_2O (1:2));• Depth monitored via EEG, respiration, body reflexes<br>In vitro:• Not mentioned (brain slices)	In vitro: Implicit exclusion criteria: Brain slices with poor viability or abnormal CSD signal (not explicitly stated but implied by experimental design); No explicit success rate provided<br>In vivo: Not mentioned (no explicit success rate or exclusion criteria provided in text)	Electrophysiology:• Transient negative DC shifts (in vivo);Optical imaging:• IOS changes reflect cellular depolarization;• Quantified by gray level variations.Biochemical:• MDA elevation post-CSD (p<0.001);Immunohistochemistry:• TRPA1 in cortical neurons & astrocytes (Figs 1-2)
Won,L et al.(2020)	Microinjection of 0.5M KCl into rostral neocortex (3 mm anterior to bregma, 2 mm lateral to midline)	- Concentration: 0.5M KCl<br>- Volume: <10 nl (threshold, P3); 10–30 nl (actual operation, P11)<br>- Frequency: Every 9–10 min<br>- Total Events: 8–9 SDs over 90 min	SD induction performed 24 hours after intranasal IGF-1/vehicle pretreatment	- Type: Isoflurane (5% induction → 3% surgery → 1.5% SD recording)<br>- Monitoring:D12- SpO_2: 95–100 mmHg (pulse oximeter);- Temp: 37±0.5°C (rectal probe);- Depth: Absence of hind paw pinch withdrawal + stable respiration + pink extremities	- Success Rate: 100% (7–9 SDs/animal confirmed by DC shift);- Exclusion Criteria: Not mentioned (all animals included in analysis)	- Electrophysiology: DC shift (~10 mV negative, ~1 min duration, Fig 2)- Biomarkers:- ↑639% MDA in TG<br>- ↑395% CGRP in TG;- ↑183% c-Fos in TCC at -4.5 mm from obex (vs. sham, *p*<0.001);
Araújo,A.D.O et al.(2023)	Chemical stimulation: Topical application of 2% KCl on the exposed frontal cortical surface of the right hemisphere.	Solution: 2% KCl (~270 mM);<br>Application: Cotton ball (1–2 mm diameter) applied for 1 min.Interval: Repeated every 20 min during the 4-hour recording session	Applied every 20 min during a 4-hour electrophysiological recording session (PND 36–42).	Type: Urethane (1 g/kg) + Chloralose (40 mg/kg), intraperitoneal injection. Depth Monitoring: Rectal temperature maintained at 37±1°C; animals breathed spontaneously.	Success Rate: 10–12 CSD episodes recorded per rat (n=10/group).Exclusion Criteria: Not mentioned, but likely excluded if CSD wave propagation failed or signal quality was poor.	Validation Method: Quantified CSD propagation velocity, amplitude, and duration.Key Evidence: Significant dose-dependent changes:- Velocity: MLT-10: 2.86±0.14 mm/min vs. MLT-40: 3.96±0.16 mm/min (*p*<0.01);- Amplitude/Duration: Differential effects (Fig 5C,D).
Ackermann,M.A et al.(2024)	- K^+ microinjection: Stratum radiatum (~100 μm next to recording site in hippocampus)<br>- O_2 withdrawal: Whole slice (conditioning ACSF with 95% N_2/5% CO_2 + 2 mM Na_2SO_3)<br>- Mitochondrial uncoupling: FCCP (2 μM) in ACSF	• Stimulus: 10 pulses at 10 Hz (100 μ s/pulse) ;• Current: Incrementally<br>increased (start: half-maximal field	- Hypoxia-induced SD: 3.96 ± 0.97 min after hypoxia onset<br>- FCCP-induced SD: 7.0 ± 2.7 min after FCCP application<br>- K^+-induced SD: 3–7 s after microinjection	Decapitation under deep anesthesia (isoflurane or methoxyflurane). No depth monitoring described.	- Success rate: Not mentioned;- Exclusion: Outlier slices via ROUT test (Q=1%) based on poor;optical/electrophysiological data	Core marker: Negative DC shift (ΔV_o amplitude: -3.5 to -4.6 mV, Table 1); roGFP oxidation (cytosolic/mitochondrial)<br>Functional recovery: Reversible fEPSP suppression in K^+-SD (Fig 3B) vs. irreversible in hypoxia-SD (Fig 3A)<br>Redox dynamics: Oxidation wavefront propagation (2.11–2.61 mm/min for K^+/hypoxia/FCCP-SD, Fig 2G)<br>Specific validation: ① roGFP calibration

Study Reference	CSD measurement details	ROS indicators	Detection Sites	Time Points	Standardization
Viggiano,A et al.(2011)	- Induction: Topical application of 3M KCl on cortical surface for 20 min;- Sampling: Continuous EEG/DC recording; Microdialysis at 15-min intervals;- Mean CSD waves: 6±2; Amplitude: 8±2 mV; Duration: 2.3±0.4 min	1. Superoxide:<br>- Method: DHE oxidation to HEt fluorescence;- Function: Direct O_2^- detection;2. Hydrogen Peroxide (H_2O_2):;- Method: Microdialysis + HPLC (2,3-DHBA derivatization);- Function: Direct H_2O_2 detection.3. SOD Activity:- Method: Cerium-DAB histochemistry;- Function: Antioxidant enzyme activity	- Cerebral cortex (layers I-VI)<br>- Ipsilateral (KCl-treated) vs. Contralateral side comparison<br>- Layer IV specified for O_2^- imaging	- All endpoints: 1h post-CSD induction;- H_2O_2: Dynamic monitoring (0-60 min post-KCl in 15-min bins);- O_2^-/SOD: Terminal measurement at 60 min	Internal Controls:- Contralateral cortex for O_2^- & SOD;- Pre-CSD basal H_2O_2 level: 9.1±3.6 μM;Methods:- Image analysis: Uniform thresholding (LabVIEW software);- SOD quantitation: ROD = $\log_{10}$(256/gray level);- Statistical: Paired t-test (ipsilateral vs. contralateral);- Sample size: n=5 per group
Grinberg,Y.Y et al.(2012)	Stimulus protocol:10 pulses, 10 Hz (100 μs/pulse)<br>Threshold determination:Progressive current increase (start: half-maximal fEPSP current);Sampling rate:≥2 Hz (inferred from 1 Hz high-pass filter)	-Primary ROS probe:CellROX™ Deep Red Reagent (fixable fluorogenic probe, near-infrared fluorescence);-OS assessment method:Menadione challenge (50 μmol/L, 1 hour)	Electrophysiology:CA3 pyramidal neuron layer (genu of CA3);ROS measurement:Uniform CA3 area (1.70 mm² at 10× magnification)	IGF-1 exposure :Acute (15-30 min), 3-day, 7-day (phasic: day/night cycle);OS measurement:24 hours post-SD	Inter-experiment control:Sham control data normalized to 1.0;Imaging calibration:Acridine orange (125 μmol/L, 480/527 nm);Statistical baseline :Same-day sham/control cultures<br>Exclusion criteria:fEPSP <3 mV (at 10–20 μA stimulus)
Grinberg,Y.Y et al.(2013)	- Stimulation: 10×100 μs pulses at 10 Hz, ~1000 μA<br>- Frequency: 6 SDs/hour (every 7-9 min);- Baseline sampling: ≤0.2 Hz;- SD continuous sampling rate: Not mentioned	- Primary: CellROX™ Deep Red;- Targets: Superoxide, OH•, $ONOO^-$; weak Not mentioned;- Validation: Protein carbonyl quantification	- Region: Hippocampal CA3;- Cells: Neurons ($NeuN^+$), astrocytes ($GFAP^+$),oligodendrocytes (RIP^+), microglia (Isolectin GS-$IB4^+$)	- OS: 24h post-SD;- TNF-α: 6h post-SD;- SD threshold: Baseline + 3d post-SD	- Vitality: Sytox Green (21 DIV);- Controls: Antibody/lectin omission;- Thresholding: Equal for sham/SD;- Normalization:• OS/TNF-α: Sham=1.0;• TNF-α: Total protein (MicroBCA)
Shatillo,A et al.(2013)	- Sampling rate: 50 kHz (spike recording);- fMRI TR: 2 s (1800 volumes in 1 h);- Induction: 5 μl of 1 M KCl applied to dura mater;- CSD wave duration: 46.7 ± 6.4 s (LFP);- Total recording duration: 1 h	- Primary marker: Malondialdehyde (MDA);- Function: Lipid peroxidation end-product;- Detection method: TBARS assay;- Standard curve range: 0–100 μM (tetramethoxypropane)	- Cortex (ipsilateral, fMRI-mapped);- Meninges (dura mater)<br>- Bilateral trigeminal ganglia;- Functional tests: Isolated hemiskull (trigeminal nerves)	- Biochemical sampling: After 1-h CSD recording + cardiac perfusion;- Stimulation duration: 15 min (H_2O_2/capsaicin for CGRP release)	- Protein normalization: μmol MDA/mg protein (BioRad assay, Lowry method)<br>- Controls: Sham surgery (saline instead of KCl) + contralateral tissues<br>- Baseline electrical activity: Pre-stimulus spike rate<br>- Baseline CGRP: Unstimulated neurons (25±5.9 pg/ml)<br>- Spike detection criteria: Duration 0.3–3 ms, amplitude >4 SD
Malkov,A et al.(2014)	Induction: Topical 3M KCl on parieto-occipital cortex; Recording: DC field potentials via ACSF-filled glass microelectrode (depth 0.5 mm) in frontal cortex; Sampling rate: ≥100 Hz (implied by 0.1 Hz high-pass filtering); Quantification: CSD event frequency (events/hour); KCl replacement: every 30 minutes	CellROX® Orange (5 μmol/L); Function: General cytoplasmic ROS sensor; Detection: Fluorescence intensity (ex 548 nm / em 632 nm LP); Loading: 45 min incubation with 0.05% pluronic acid	Hippocampal slices (stratum pyramidale/granulare, 350 μm thickness); In vivo: Parieto-occipital cortex (KCl application), frontal cortex (recording), lateral ventricle (Tempol injection coordinates: 0.7 mm posterior to bregma, 1 mm lateral)	Baseline: Pre-stimulation after 2 hr ACSF recovery; ROS baseline: ≥45 min post-dye loading; Post-stimulus: 0-30 min (reversible MC) or >30 min (irreversible MC); Irreversible MC proportion: 50% of cases	ROS: $\Delta F/F\% = [(F-F_0)/F_0]\times 100$ (F_0: 2,000-3,000 intensity units); O_2: Two-point calibration at 33°C (95% O_2 solution = 714 Torr, ambient air = 155 Torr); K^+: Pre-experiment calibration (42 mV/decade sensitivity)
Pusic,K.M et al.(2014)	• Sampling rate: Not mentioned (Digidata 1322A ADC board used; typical capability 250 kHz)<br>• Stimulation: - Synaptic: 10 pulses at 10 Hz (100 μs/pulse) ;- KCl: 1M KCl-agar wand (1–2 s contact) ;• Recording: Interstitial DC potentials	CellROX Deep Red: General oxidative stress probe (fluorogenic) :• ThiolTracker: Reduced thiols (primarily glutathione) ;• Protein carbonyl content: Spectrophotometric assay (ROS-mediated protein damage) ;• iNOS mRNA: RT-PCR (M1 marker, produces RNS)	• Location: - Electrophysiology/ROS: CA3 stratum pyramidale :- Protein carbonyl: Neocortex (whole animal)	• Time points: - Baseline: Untreated controls ;- Post-treatment: - Clodronate: 7 days ;- IL-11/Minocycline: 3 days;- Post-SD: Immediate (electrophysiology), 2h (CellROX + menadione)	• Immunostaining: - Quantification: Fixed thresholding via MetaMorph v7.5.4.0;• RT-PCR: Endogenous control Rp113a (delta-delta Ct method) ;• Protein carbonyl: Normalized to total protein (BCA assay) ;• ROS imaging: Identical CA3 region, standardized optical intensity
Pusic,A.D et al.(2015a)	Stimulation: 10 pulses at 10 Hz (100 μs/pulse), 1000 μA current<br>Recording system: Axoprobe A1 + Digidata 1440A	CellROX Deep Red (fluorogenic OS probe)<br>Protein carbonyl content (Abcam kit #ab126287)<br>ThiolTracker (reduced thiols/GSH probe)	In vitro: CA3 pyramidal neuron layer<br>In vivo: Paramedian neocortex (gray matter)<br>& Lateral corpus callosum (white)	SD effects: 1, 3, 7 days post-SD;Acute OS: 1h after IFNγ + CellROX incubation;T cell/IFNγ in vivo: 1 day post-SD	Western blot: β-actin;qPCR: Ribosomal protein L13a (Rp113a);IHC Quantification: Integrated optical density;nsMase2 Activity: Units/mg protein;In vivo control: Contralateral neocortex
Pusic,A.D et al.(2015b)	Stimulation Protocol：- In vitro: 10 pulses at 10 Hz (100 μs/pulse), current from 10 to 10,000 nC<br>- In vivo: KCl microinjection (0.5 M, 8-12 μm tip)<br>Recording Method：DC potential shift recorded via 2-4 μm microelectrode (750 μm depth)<br>Threshold Definition：Minimal charge (nC) to induce SD in CA3 pyramidal layer; 10,000 nC set as max threshold	1. CellROX Deep Red: Total ROS (co-incubated with menadione 8.6 μg/mL) ;2. Protein Carbonyls (Abcam #ab12687): Oxidized proteins ;3. ThiolTracker: Total reduced thiols (mainly GSH)	In Vitro：Hippocampal slice cultures: Standardized CA3 stratum pyramidale area<br>Exosome Source:Microglia (confirmed by isolectin-GS-IB4 staining)	- IFNγ effects: 3 & 7 days post-treatment (optimal at 7d);- Exosome effects: 3h (OS) & 7d (MBP/SDT/GSH);- Nasal IFNγ: 1 & 3 days	- Age-matched untreated cultures;- Sham nasal administration (succinate buffer);- Exosomes from unstimulated microglia;- qPCR: ΔΔCt with Rp113a;- FluoroMyelin intensity: Integrated optical density;- Protein quantification: β-actin for WB;qPCR significance: Fold-change >2;ANOVA with Holm-Sidak post hoc;Power (1-β) >0.8
Pusic,K.M et al.(2019)	- Stimulation protocol: 10 pulses at 10 Hz (100 μs/pulse) starting at half-maximal field potential current, increased every 3 min<br>- Sampling rate: Not mentioned (Digidata 1422A used)	- Protein carbonyl: Direct marker of protein oxidation (measured by DNPH assay);- iNOS mRNA: Marker of M1 microglial polarization (indirect ROS source via Not mentioned)	- In vitro: Hippocampal slice cultures (CA region)<br>- In vivo: Neocortex (SD threshold & protein carbonyl), Whole brain (iNOS mRNA)	- SD threshold (in vitro): 3 days post-exosome treatment;- SD threshold (in vivo): 1 day post-nasal administration;- iNOS mRNA: 1 day post-administration;- Protein carbonyl: 3 days post-administration	- Protein carbonyl: Quantified by BCA assay with streptozotocin pre-treatment (remove nucleic acids);- RT-qPCR: Normalized to Rpl13a using ΔCt method;- Electrophysiology: Threshold values normalized to sham group = 1.0
Jiang, L et al.(2019)	Sampling rate: 10 Hz (EEG & DC potentials)；CSD parameters: latency, magnitude (AUC), number of waves	Malondialdehyde (MDA)	Ipsilateral cerebral cortex (rat);Somatosensory cortex layers 4–6 (mouse)	Immediately after CSD induction (rat MDA);45-min interval between CSD episodes (mouse)	Total protein normalization (BCA kit) for MDA;Baseline normalization: 2nd vs 1st CSD in slices
Won,L et al.(2020)	Induction: 0.5 M KCl microinjection (8–30 nL, 20 psi, every 9–10 min)<br>• Recording: Extracellular DC shift via micropipette (4μm tip)<br>• Sampling rate: Not specified (equipment: Axoprobe A1 amplifier + Axon 1440A ADC)	Malondialdehyde (MDA)<br>• Function: Lipid peroxidation end product (sole oxidative stress marker quantified)	1. Trigeminal ganglion (V1 ophthalmic division)<br>2. Trigeminocervical complex (laminae I–II, –4.5 mm from obex)<br>3. Blood plasma (CGRP only)	mmediately after last SD (90-min induction protocol)	• Paired processing: Sham/experimental sections stained together;• Image analysis: Fixed camera gain/threshold, integrated optical intensity ;• Normalization: Natural log-ratios (Exp/Sham) ;• Internal control: Sham-operated animals (no housekeeping protein)
Araújo,A.D.O et al.(2023)	Velocity: Calculated from time for CSD wave to propagate between two parietal electrodes<br>- Amplitude/Duration: Measured from negative component of DC slow potential shift<br>- Stimulus: 2% KCl applied topically to frontal cortex (1 min every 20 min)<br>- Recording: Ag-AgCl-Agar-Ringer electrodes at parietal cortex	- MDA: Malondialdehyde (lipid peroxidation marker; nmol/mg protein)<br>- SOD: Superoxide dismutase (antioxidant enzyme activity; U SOD·min^{-1}/mg protein)	- Behavior: Open field (89cm diameter) & elevated plus maze (49x10cm arms)<br>- Electrophysiology: Frontal cortex (stimulus), Parietal cortex (recording)<br>- Biochemistry: Cerebral cortex tissue homogenate	- MLT administration: PND7-27 (alternate days; 11 doses);- Behavioral tests: PND30-34;- CSD recording: PND36-42;- Biochemistry: Immediately post-CSD (PND36-42)	- Biochemistry: BCA protein assay (MDA/SOD per mg protein);- Behavior: ANY-maze™ video tracking software (v4.99m);- CSD velocity: Fixed inter-electrode distance (implicit spatial normalization)
Ackermann,M.A et al.(2024)	- Sampling rate:<br>· DC potential: 100 Hz<br>· fEPSPs: 20 kHz<br>- Electrode: Glass microelectrode (~5 MΩ) in CA1 stratum radiatum	- Name: roGFP1 (cytosolic/mitochondrial)<br>- Function: · Excitation-ratiometric (395 nm/475 nm) ;· Reversible redox reporter	- Primary: CA1 stratum pyramidale (soma layer) ;- ROI size: ~20×40 μm (multi-neuron level)	Baseline Stabilization：≥15 min in oxygenated ACSF;Drug Pre-treatment：15 min (e.g., DPI, allopurinol);Optical Sampling：Standard: Every 10 s (0.1 Hz) ;Validation: Every 3 s (0.33 Hz) SD Onset Definition=0t=0 = Negative DC deflection (ΔVsΔVs);Oxidation Analysis• At peak oxidation OR 300 s post-SD onset;Wash-out Start• Hypoxia/FCCP: 3 min post-SD;K^+-SD: After ΔVsΔVs recovery;H_2O_2/DTT Calibration7 min exposure (each)	- Calibration: E14· Full oxidation: 5 mM H_2O_2 (7 min) ; · Full reduction: 10 mM DTT (7 min) ;- Controls: · Autofluorescence: 25× lower vs. roGFP (Fig. 1H) ;· DMSO: ≤ 0.2% (no redox effect) F5;· Illumination: 1,000 cycles (ΔOxD = 2.43±2.44%)

Study Reference	CSD Results	ROS Results	CSD-ROS correlation	Correlation Coefficient
Viggiano,A et al.(2011)	Number of CSD waves: 6±2; Mean amplitude of CSD waves: 8±2 mV; Mean duration of CSD waves: 2.3±0.4 min. No CSD waves were observed in the contralateral cortex, with stable DC potential and normal EEG activity maintained.	1. Superoxide anion (O_2^-): The concentration of O_2^- in the ipsilateral cerebral cortex was significantly decreased at 1 h after CSD induction (compared with the contralateral side: number of O_2^--positive neurons, t_s=2.73, P<0.05; above-threshold proportional area of fluorescence, t_s=2.79, P<0.05).<br>2. Hydrogen peroxide (H_2O_2): The basal level of H_2O_2 was 9.1±3.6 μM. The H_2O_2 level in the ipsilateral cortex increased significantly at 15 min after KCl application for CSD induction and remained elevated up to 60 min post-induction. Repeated measures ANOVA revealed significant effects of time [F(4,50)=10.3, P<0.01], treatment [F(1,50)=27.99, P<0.01], and time × treatment interaction [F(4,50)=8.22, P<0.01], with no obvious changes in the contralateral cortex.<br>3. Superoxide dismutase (SOD) activity: SOD activity in the ipsilateral cerebral cortex was significantly increased at 1 h after CSD induction (compared with the contralateral side, t_s=7.43, P<0.01).	N/A	Not mentioned
Grinberg,Y.Y et al.(2012)	Acute IGF-1: 24-fold ↑ vs control (p=0.001)<br>3-day IGF-1: 75-fold ↑ (p<0.001)<br>7-day IGF-1: 22-fold ↑ (p<0.001)	Acute IGF-1: 20% ↓ in SD-induced OS vs SD-only<br>3-day IGF-1: 30% ↓<br>7-day IGF-1: 73% ↓ (p<0.001 vs SD-only)	Not statistically tested<br>Indirect evidence: H_2O_2 ↓ SD threshold, Ascorbate ↑ SD threshold, IGF-1 reverses oxidant effects	Not mentioned
Shatillo,A et al.(2013)	Number of CSD waves: 3.8 ± 1.6 per animal (mean ± SEM)<br>Duration of CSD: 46.7 ± 6.4 s (mean ± SEM)<br>Amplitude of CSD: 31.2 ± 6.2 mV (mean ± SEM)<br>Latency to first wave: 114.2 ± 60.8 s (mean ± SEM)<br>Inter-wave interval: 427.6 ± 302.8 s (mean ± SEM)	Cortical MDA (ipsilateral): Significantly elevated vs. contralateral/sham<br>Meningeal MDA (ipsilateral): Significantly elevated vs. contralateral/sham<br>Trigeminal ganglia MDA: Bilateral elevation vs. sham controls	Not mentioned	Not mentioned
Grinberg,Y.Y et al.(2013)	-TNF-α effect：Control: 1.00 ± 0.10; TNF-α: 0.52 ± 0.03 (p=0.002)<br>- SD self-primingBaseline: 1.00 ± 0.10; Post-SD: 0.15 ± 0.03 (p<0.001p<0.001)	- Astrocytes (SD vs. Sham)Sham: 1.00 ± 0.28; SD: 7.55 ± 1.70 (p=0.019p=0.019);- Astrocytes (SD + IGF-1)SD: 1.00 ± 0.11; SD+IGF-1: 0.85 ± 0.28 (p=0.65p=0.65);- Microglia (SD vs. Sham)Sham: 1.00 ± 0.22; SD: 16.27 ± 1.34 (p=0.018p=0.018);- Microglia (SD + IGF-1)SD: 1.00 ± 0.24; SD+IGF-1: 0.08 ± 0.02 (p=0.018p=0.018);- Microglia (TNF-α effect)Control: 1.00 ± 0.19; TNF-α: 18.25 ± 6.22 (p=0.005p=0.005);-Microglia (TNF-α + IGF-1)：TNF-α+IGF-1: 1.92 ± 0.49 (vs. TNF-α alone)	- TNF-α protein after SDSham: 1.00 ± 0.04; SD: 1.62 ± 0.20; SD+IGF-1: 0.71 ± 0.13 (p=0.01p=0.01);- Microglial OS + sTNFR1SD: 1.00 ± 0.27; SD+sTNFR1: 0.13 ± 0.03 (p=0.036p=0.036);- SD threshold + sTNFR1SD control: 1.00 ± 0.10; SD+sTNFR1: 345 ± 29(p<0.001p<0.001);- Oxidized proteins (SD)Sham: 1.00 ± 0.08; SD: 1.38 ± 0.17 (p=0.039p=0.039);- Oligodendrocyte OSNo significant change (p=0.96p=0.96)	Not mentioned
Malkov,A et al.(2014)	KCl-induced SD frequency: Control: 0.68 events/min ;Tempol: 0.14 events/min	ROS transient amplitude: Pyruvate-ACSF: 0.80 ± 0.19% ΔF/F;Glucose-ACSF: 0.30 ± 0.07% ΔF/F	Not mentioned	Not mentioned
Pusic,K.M et al.(2014)	EE：9.82 ± SEM;NE：1.00 ± SEM;IL-11 (in vitro): 120.00 ± SEM;Control: 1.00 ± SEM;IL-11 (nasal): 24.40 ± SEM;Sham: 1.00 ± SEM;Clodronate: 0% SD induction<br>Control: 100% SD induction;M2a-CM: 349.50 ± SEM;M1-CM: 1.03 ± SEM	EE：7.10 ± SEM nmol/mg;NE：11.90 ± SEM nmol/mg;IL-11: 7.29 ± SEM nmol/mg;Sham: 13.89 ± SEM nmol/mg;M1-CM: 1.33 ± SEM;M2a-CM: 1.10 ± SEM;Control: 1.00 ± SEM	N/A	Not mentioned
Pusic,A.D et al.(2015a)	Myelin basic protein levels (Western blot)：Control: 1.00 ± 0.05 (n=14);1d post-SD: 0.54 ± 0.05 (n=5);7d post-SD: 1.00 ± 0.04 (n=18);Myelin disruption (Electron microscopy)：Control: 1.00 ± 0.22;1d post-SD: 2.53 ± 0.02;7d post-SD: 1.14 ± 0.11	• ROS (CellROX) Control: 1.00 ± 0.07;IFNγ-treated: 1.50 ± 0.27 (n=5);• Glutathione (ThiolTracker) Control: 1.00 ± 0.08 (n=12);IFNγ-exposed: 0.66 ± 0.07 (n=9)	-T cell lymphocyte depletion vs. Loss of myelin basic protein：Anti-CD4 + SD: MBP = 0.87±0.08；vs SD alone: 0.54±0.05<br>-nsMase2 inhibition vs MBP protection：GW4869 + SD: MBP=1.30±0.11 ；vs SD alone: 0.54±0.05<br>-cooperative effect of IFNγ and TNFα：IFNγ: 0.70±0.10；IFNγ +TNFα: 0.32±0.05	Not mentioned
Pusic,A.D et al.(2015b)	In Vitro Control (pulsed IFNγ): 1.00 ± 0.65 nC;In Vitro 7-day phasic IFNγ: 605 ± 121 (relative to control);In Vivo Nasal IFN γ (1-day): 63.4 ± 4.58 (relative to sham)	In Vitro Control: 1.00 ± 0.03 (relative fluorescence);In Vitro Pulsed IFNγ (7-day): 0.69 ± 0.02 (relative);In Vivo Protein Carbonyl (Sham): 12.96 ± 0.96 nmol/mg;In Vivo Protein Carbonyl (IFNγ): 8.68 ± 1.73 nmol/mg;Glutathione (GSH) ;In Vitro Control: 1.00 ± 0.06 (relative ThiolTracker);In Vitro Exosome-treated: 1.82 ± 0.09 (relative)	Not mentioned	"- Hypothesis Testing:t-test, ANOVA + Holm-Šidák post hoc<br>- Non-parametric Alternatives Mann-Whitney U / Kruskal-Wallis<br>- Data Transformation Fold change (controls = 1.0)"
Pusic,K.M et al.(2019)	In vitro (Slice cultures):Control: 1.00 ± 0.45 (n=8)<br>IFNγ-DC-Exos: 12.5 ± 1.52 (n=8);In vivo (Rats):Sham (Vehicle): 1.00 ± 0.14 (n=3–5);IFNγ-DC-Exos: 57.0 ± 4.27 (n=3–5)	Protein carbonyl content:Sham: 7.25 ± 0.83 (n=7);IFNγ-DC-Exos: 4.58 ± 0.55 (n=5);iNOS mRNA;Sham: 1.00 (reference);IFNγ-DC-Exos: 4.37-fold decrease (no SEM/p-value)	No (no statistical correlation between CSD and ROS analyzed)	In vitro CSD: p ≤ 0.001 (Holm-Sidak)<br>- In vivo CSD: p ≤ 0.001 (Holm-Sidak)<br>- Protein carbonyl: *p* = 0.035 (Holm-Sidak)<br>- iNOS: No p-value (significance by >2-fold change)<br><br>Statistical Methods - Normality test: p<0.05 reject (method unspecified)<br>- Group comparison: ANOVA + Holm-Sidak post hoc<br>- Data expression: Mean ± SEM<br>- No data transformation mentioned
Jiang, L et al.(2019)	- In vivo (rats) CSD number: 6 [3] (median [range])<br>Latency: 2.4 (1.2) min (median (range))<br>Magnitude: 6.5 (9.5) mV·min (median (range))<br>In vitro (mouse slices) Submaximal CSD latency: 18.3 [15] s (median [range])<br>Magnitude: 61.5% (range: 55.48%) (median (range), vs 1st episode)	- MDA level (rats) Sham group: 0.64 (0.29) μmol/mg (median (range))<br>CSD group: 1.07 (0.7) μmol/mg (median (range))	Yes (CSD magnitude vs MDA level)	r = 0.5135 (95% CI Not mentioned)
Won,L et al.(2020)	Mean number of SD events:8.4 ± 0.4 SDs (mean ± SEM)<br>CSD-induced MDA increase (Ln ratio):Ln(SD/sham) = 2.00 ± 0.190 → 639% increase vs ND (p<0.001, power=1.00)<br>CSD-induced CGRP increase (Ln ratio):Ln(SD/sham) = 1.60 ± 0.31 → 395% increase vs ND (p<0.001, power=1.00)<br>CSD-induced c-fos increase (Ln ratio):Ln(SD/sham) = 1.04 ± 0.20 → 183% increase vs ND (p<0.001, power=1.00)	Naive IGF-1 effect on CGRP (Ln ratio):Ln(IGF-1/sham) = -1.66 ± 0.32 → 81% decrease vs ND (p<0.001, power=1.00)<br>SD+IGF-1 effect on MDA (Ln ratio):Ln(IGF-1/sham) = -1.76 ± 0.62 → 83% decrease in MDA (p<0.001, power=1.00)<br>SD+IGF-1 effect on CGRP (Ln ratio) :Ln(IGF-1/sham) = -0.90 ± 0.15 → 59% decrease in CGRP (p<0.001, power=1.00)<br>SD+IGF-1 effect on c-fos (Ln ratio):Ln(IGF-1/sham) = -0.60 ± 0.06 → 45% reduction in c-fos (p<0.001, power=0.97)	Reported correlation?:No direct correlation analysis between CSD and ROS parameters<br>Statistical Method:Ln(ratio) vs 0 by t-test (two-tailed)<br>Data Transformation:All immunostaining data as Ln(experimental/sham ratio)	Not mentioned
Araújo,A.D.O et al.(2023)	- Velocity: Intact: 3.31±0.10 mm/min; Vehicle: 3.25±0.11 mm/min; MLT-10: 2.86±0.14 mm/min; MLT-40: 3.96±0.16 mm/min<br>- Amplitude: Intact: 8.9±2.4 mV; Vehicle: 9.0±1.7 mV; MLT-10: 6.8±1.2 mV; MLT-40: 13.2±2.3 mV<br>- Duration: Intact: 83.9±5.4 s; Vehicle: 82.1±4.0 s; MLT-10: 96.5±9.1 s; MLT-40: 70.7±4.0 s	- MDA: Intact: 6.21±0.58 nmol/mg protein; Vehicle: 6.93±2.53 nmol/mg protein; MLT-10: 3.34±1.24* nmol/mg protein; MLT-40: 4.32±0.82 nmol/mg protein<br>- SOD: Intact: 7.39±3.88 AU/min/mg protein; Vehicle: 7.59±3.71 AU/min/mg protein; MLT-10: 19.49±4.57** AU/min/mg protein; MLT-40: 11.00±3.33 AU/min/mg protein	Not mentioned. Mechanistic link implied: MLT-10 showed ↓MDA + ↑SOD → decelerated CSD + reduced anxiety	Not mentioned
Ackermann,M.A et al.(2024)	-Normoxic K^+-induced SD:ΔV_o = -4.60 ± 2.35 mV; t½ = 91.52 ± 72.38 s;-Hypoxia-induced SD (HSD):ΔV_o = -3.54 ± 1.21 mV;Δt = 3.96 ± 0.97 min;-FCCP-induced SD:ΔV_o = -4.55 ± 1.76 mV;Δt = 7.01 ± 2.65 min;-Ca^{2+}-free + K^+-SD:ΔV_o = -3.13 ± 1.51 mV;t½ = 40.14 ± 19.61 s;-DPI + K^+-SD:ΔV_o = -5.02 ± 2.32 mV;-Mitochondrial oxidation (HSD):N/A	-Normoxic K^+-induced SD:ΔOxD_{roGFPc} = +5.6% ± 2.5%;Wavefront velocity = 2.52 ± 0.47 mm/min;-Hypoxia-induced SD (HSD):ΔOxD_{roGFPc} = +9.8% ± 1.1%;Wavefront velocity = 2.61 ± 0.41 mm/min;-FCCP-induced SD:ΔOxD_{roGFPc} = +5.4% ± 0.6%;Wavefront velocity = 2.11 ± 0.47 mm/min;-Ca^{2+}-free + K^+-SD:ΔOxD_{roGFPc} = +2.13% ± 0.73%;Wavefront velocity = 1.74 ± 0.37 mm/min;-DPI + K^+-SD:Δ OxD_{roGFPc} = +8.30% ± 1.72%;-Mitochondrial oxidation (HSD):Baseline OxD_{roGFPm} = 67.3% ± 6.7%;ΔOxD_{roGFPm} = +1.42% ± 1.50%	Not mentioned	Not mentioned
