# Supplementary material for: Compartment-specific redox modulation in migraine with aura: a systematic review of context-dependent mechanisms
Source: J Oral Facial Pain Headache. 2026 Sep 12;40(5):39–53. doi: 10.22514/jofph.2026.059 (PMC13678554; doi:10.22514/jofph.2026.059)
Supplement: Supplementary file 3 [file Supplementary-material-2.docx]

Supplementary material 2

**Detailed Search Strategies**

Systematic Review Title: Compartment-specific redox modulation in migraine with aura: a systematic review of context-dependent mechanisms

Databases Searched: PubMed (via MEDLINE), Embase, Web of Science Core Collection, CNKI (China National Knowledge Infrastructure), Wanfang Database.

Search Date: From inception to June 2025

The table below details the complete electronic search strategies employed for each database.

Database Search Strategy:

Domain 1-Migraine

Medical Subject Headings (MeSH): migraine

Text Words: episodic migraine

chronic migraine

migraine with aura

migrain* with aura

aura* migrain*

classical migrain*

hemiplegic migrain*

basilar migrain*

retinal migrain*

Domain 2-Cortical Spreading Depression

Medical Subject Headings (MeSH): cortical spreading depression

Text Words: spreading depression

CSD

cortical depolarization

Leão depression

Leao depression

Domain 3-Oxidative Stress/Redox

Medical Subject Headings (MeSH): oxidative stress

Text Words: oxidat* stress

free radical*

reactive oxygen species

ROS

lipid peroxidation

oxidat* damage

antioxidant*

redox imbalance

redox

oxidative eustress

oxidative distress

nitrosative stress

Domain 4-Animal/Rodent Models

Medical Subject Headings (MeSH): animals

Text Words: animal*

rodent*

rat

rats

mouse

mice

*in vivo*

*in vitro*

Sprague Dawley

Wistar

C57BL

PubMed:

((migraine[Title/Abstract] OR episodic migraine[Title/Abstract] OR chronic migraine[Title/Abstract] OR migraine with aura[Title/Abstract] OR migrain* with aura[Title/Abstract] OR aura* migrain*[Title/Abstract] OR classical migrain*[Title/Abstract] OR hemiplegic migrain*[Title/Abstract] OR basilar migrain*[Title/Abstract] OR retinal migrain*[Title/Abstract]) AND (cortical spreading depression[Title/Abstract] OR spreading depression[Title/Abstract] OR CSD[Title/Abstract] OR cortical depolarization[Title/Abstract] OR Leão depression[Title/Abstract] OR Leao depression[Title/Abstract]) AND (oxidative stress[Title/Abstract] OR oxidat* stress[Title/Abstract] OR free radical*[Title/Abstract] OR reactive oxygen species[Title/Abstract] OR ROS[Title/Abstract] OR lipid peroxidation[Title/Abstract] OR oxidat* damage[Title/Abstract] OR antioxidant*[Title/Abstract] OR redox imbalance[Title/Abstract] OR redox[Title/Abstract] OR oxidative eustress[Title/Abstract] OR oxidative distress[Title/Abstract] OR nitrosative stress[Title/Abstract]) AND (animal*[Title/Abstract] OR rodent*[Title/Abstract] OR rat[Title/Abstract] OR rats[Title/Abstract] OR mouse[Title/Abstract] OR mice[Title/Abstract] OR *in vivo*[Title/Abstract] OR *in vitro*[Title/Abstract] OR Sprague Dawley[Title/Abstract] OR Wistar[Title/Abstract] OR C57BL[Title/Abstract]))

Embase:

(‘migraine’/exp OR ‘episodic migraine’:ab,ti OR ‘chronic migraine’:ab,ti OR ‘migraine with aura’:ab,ti OR ‘migrain* with aura’:ab,ti OR ‘aura* migrain*’:ab,ti OR ‘classical migrain*’:ab,ti OR ‘hemiplegic migrain*’:ab,ti OR ‘basilar migrain*’:ab,ti OR ‘retinal migrain*’:ab,ti) AND (‘cortical spreading depression’/exp OR ‘spreading depression’:ab,ti OR ‘csd’:ab,ti OR ‘cortical depolarization’:ab,ti OR ‘leão depression’:ab,ti OR ‘leao depression’:ab,ti) AND (‘oxidative stress’/exp OR ‘oxidat* stress’:ab,ti OR ‘free radical*’:ab,ti OR ‘reactive oxygen species’:ab,ti OR ‘ros’:ab,ti OR ‘lipid peroxidation’:ab,ti OR ‘oxidat* damage’:ab,ti OR ‘antioxidant*’:ab,ti OR ‘redox imbalance’:ab,ti OR ‘redox’:ab,ti OR ‘oxidative eustress’:ab,ti OR ‘oxidative distress’:ab,ti OR ‘nitrosative stress’:ab,ti) AND (‘animal’/exp OR ‘rodent’/exp OR ‘rat’:ab,ti OR ‘rats’:ab,ti OR ‘mouse’:ab,ti OR ‘mice’:ab,ti OR ‘*in vivo*’:ab,ti OR ‘*in vitro*’:ab,ti OR ‘sprague dawley’:ab,ti OR ‘wistar’:ab,ti OR ‘c57bl’:ab,ti)

Web of Science:

(TS = (migraine) OR TS = (“episodic migraine”) OR TS = (“chronic migraine”) OR TS = (“migraine with aura”) OR TS = (“migrain* with aura”) OR TS = (“aura* migrain*”) OR TS = (“classical migrain*”) OR TS = (“hemiplegic migrain*”) OR TS = (“basilar migrain*”) OR TS = (“retinal migrain*”)) AND (TS = (“cortical spreading depression”) OR TS = (“spreading depression”) OR TS = (CSD) OR TS = (“cortical depolarization”) OR TS = (“Leão depression”) OR TS = (“Leao depression”)) AND (TS = (“oxidative stress”) OR TS = (“oxidat* stress”) OR TS = (“free radical*”) OR TS = (“reactive oxygen species”) OR TS = (ROS) OR TS = (“lipid peroxidation”) OR TS = (“oxidat* damage”) OR TS = (antioxidant*) OR TS = (“redox imbalance”) OR TS = (redox) OR TS = (“oxidative eustress”) OR TS = (“oxidative distress”) OR TS = (“nitrosative stress”)) AND (TS = (animal*) OR TS = (rodent*) OR TS = (rat) OR TS = (rats) OR TS = (mouse) OR TS = (mice) OR TS = (“*in vivo*”) OR TS = (“*in vitro*”) OR TS = (“Sprague Dawley”) OR TS = (Wistar) OR TS = (C57BL))

CNKI search strategy (Chinese):

(SU = ‘偏头痛’ OR SU = ‘发作性偏头痛’ OR SU = ‘慢性偏头痛’ OR SU = ‘伴先兆偏头痛’ OR SU = ‘先兆性偏头痛’ OR SU = ‘典型偏头痛’ OR SU = ‘偏瘫性偏头痛’ OR SU = ‘基底型偏头痛’ OR SU = ‘视网膜性偏头痛’) AND (SU = ‘皮质扩散性抑制’ OR SU = ‘皮层扩散性抑制’ OR SU = ‘扩散性抑制’ OR SU = ‘CSD’ OR SU = ‘皮质去极化’ OR SU = ‘Leão抑制’) AND (SU = ‘氧化应激’ OR SU = ‘氧化还原’ OR SU = ‘自由基’ OR SU = ‘活性氧’ OR SU = ‘ROS’ OR SU = ‘脂质过氧化’ OR SU = ‘氧化损伤’ OR SU = ‘抗氧化’ OR SU = ‘氧化还原失衡’ OR SU = ‘亚硝化应激’ OR SU = ‘氧化性应激’ OR SU = ‘氧化还原应激’) AND (SU = ‘动物’ OR SU = ‘大鼠’ OR SU = ‘小鼠’ OR SU = ‘啮齿类’ OR SU = ‘体内’ OR SU = ‘体外’ OR SU = ‘实验动物’)

Wanfang search strategy (Chinese):

主题:(偏头痛 OR 发作性偏头痛 OR 慢性偏头痛 OR 先兆性偏头痛 OR 无先兆偏头痛 OR 经典型偏头痛 OR 偏瘫型偏头痛 OR 基底动脉型偏头痛 OR 视网膜性偏头痛) AND 主题:(皮质扩散性抑制 OR 皮层扩散性抑制 OR 扩散性去极化 OR CSD OR Leão抑制) AND 主题:(氧化应激 OR 氧化还原 OR 自由基 OR 活性氧 OR ROS OR 脂质过氧化 OR 氧化损伤 OR 抗氧化剂 OR 抗氧化 OR 氧化还原失衡 OR 亚硝化应激 OR SOD OR MDA) AND 主题:(动物 OR 大鼠 OR 小鼠 OR 啮齿类 OR 体内 OR 体外 OR 实验动物)
