# Supplementary material for: Compartment-specific redox modulation in migraine with aura: a systematic review of context-dependent mechanisms
Source: J Oral Facial Pain Headache. 2026 Sep 12;40(5):39–53. doi: 10.22514/jofph.2026.059 (PMC13678554; doi:10.22514/jofph.2026.059)
Supplement: Supplementary file 4 [file Supplementary-material-4.docx]

Supplementary material 4

| Study Reference | Sequence generation | Baseline characteristics | Allocation concealment | Random housing | Performance bias | Random outcome assessment | Detection bias | Incomplete outcome data | Selective outcome reporting | Other sources of bias |
| --- | --- | --- | --- | --- | --- | --- | --- | --- | --- | --- |
| Pusic KM *et al*. [23] (2014) | U | L | U | L | U | U | U | L | L | L |
| Araújo ADO *et al*. [18] (2023) | U | L | U | L | L | U | U | L | L | L |
| Jiang L *et al*. [15] (2019) | U | U | U | L | U | U | U | L | L | L |
| Pusic AD *et al*. [24] (2015a) | U | U | U | U | U | U | U | L | L | H |
| Grinberg YY *et al*. [25] (2013) | U | U | U | L | U | U | U | L | L | H |
| Pusic KM *et al*. [26] (2019) | U | L | U | L | U | U | U | L | L | L |
| Pusic AD *et al*. [27] (2015b) | U | L | U | L | U | U | U | L | L | H |
| Ackermann MA *et al*. [13] (2024) | U | U | U | L | U | U | U | L | L | L |
| Grinberg YY *et al*. [28] (2012) | U | L | U | L | U | U | U | L | L | L |
| Won L *et al*. [29] (2020) | U | U | U | L | L | L | L | L | L | L |
| Malkov A *et al*. [14] (2014) | U | L | U | L | U | U | U | L | L | L |
| Viggiano A *et al*. [30] (2011) | U | L | U | L | U | U | U | L | L | L |
| Shatillo A *et al*. [31] (2013) | U | U | U | U | U | U | U | L | L | L |

L: low risk; H: High risk; U: unclear.

Pusic KM *et al*. [23] (2014)

| Evaluation Item | Risk Assessment | Supporting Evidence |
| --- | --- | --- |
| Sequence generation | Unclear | Stated group allocation only, but method of random sequence generation not described: “Twelve male Wistar rats were group housed...whereas 12 aged-matched nonenriched (NE) rats...” |
| Baseline characteristics | Low | No baseline electrophysiological difference between EE and NE groups; SD induction after grouping (meets interventional study criteria) |
| Allocation concealment | Unclear | Not mentioned |
| Random housing | Low | Uniform baseline conditions: “Sufficient space was provided to prevent dominant male behavior”; “EE rats housed in Marlau-style cage...NE rats single-housed under standard conditions” |
| Performance bias | Unclear | “Not mentioned” |
| Blinding outcome assessment | Unclear | Not mentioned: “SD threshold was determined... using a Digidata 1322A analog-digital conversion board” |
| Detection bias | Unclear | Not mentioned: “Electrophysiological data was transferred from Axon Instruments software to OriginPro...” |
| Incomplete outcome data | Low risk | Complete sample size reported for all experiments with no missing data: “all animals were included in the analysis” |
| Selective outcome reporting | Low risk | All predefined primary outcomes were fully reported: “SD threshold, protein carbonyl, Iba1 staining, qPCR data” |
| Other sources of bias | Low risk | shows no evidence of contamination, funder influence, or unit-of-analysis errors. |

Araújo ADO *et al*. [18] (2023)

| Evaluation Item | Risk Assessment | Supporting Evidence |
| --- | --- | --- |
| Sequence generation | Unclear | Not mentioned: “Each group was formed with pups from 3 to 5 distinct litters.” |
| Baseline characteristics | Low risk | Fig. 2A shows no difference in body weight between groups: “ANOVA showed no significant intergroup difference in body weight at 7 days of age ...” |
| Allocation concealment | Unclear | Not mentioned |
| Random housing | Low risk | All animals were housed under identical conditions with random assignment: “After weaning, pups were separated by sex and housed in polypropylene cages under controlled temperature (23 ℃ ± 1 ℃) with a 12–12 h light–dark cycle” |
| Performance bias | Low risk | Behavioral tests were performed at random times: “The animals were randomly tested between 8 AM and 1 PM” |
| Blinding outcome assessment | Unclear | Not mentioned: “analyzed with ANYmaze™ software; CSD propagation velocity was calculated” |
| Detection bias | Unclear | Not mentioned |
| Incomplete outcome data | Low risk | Complete sample size reported for all experiments with no missing data: “The n is 10 for each group, as stated in the methods...” |
| Selective outcome reporting | Low risk | All predefined primary outcomes were fully reported: “Behavior (anxiety/memory), CSD, MDA/SOD levels align with Introduction and Results (*e.g.*, we hypothesize that MLT differentially affects... CSD parameters”)” |
| Other sources of bias | Low risk | shows no evidence of contamination, funder influence, or unit-of-analysis errors. |

Jiang L *et al*. [15] (2019)

| Evaluation Item | Risk Assessment | Supporting Evidence |
| --- | --- | --- |
| Sequence generation | Unclear | Not mentioned: “Three groups were designed; two series of experiments were designed.” |
| Baseline characteristics | Unclear | Although the animal species, sex, and weight range were specified (“male Sprague Dawley rats, 253.8 ± 40.9 g”), between-group homogeneity of baseline characteristics (*e.g.*, weight distribution, age) was not verified: “A total of 30 male Sprague Dawley rats (253.8 ± 40.9 g)...” |
| Allocation concealment | Unclear | Not mentioned. |
| Random housing | Low risk | All animals were housed in the same environment, meeting the requirement for randomized housing: “Animals were housed in the experimental animal Centre of Soochow University...” |
| Performance bias | Unclear | Not mentioned: “Drugs were perfused into contralateral ventricle...” |
| Blinding outcome assessment | Unclear | Not mentioned: “Ipsilateral cerebral cortices... were homogenized” |
| Detection bias | Unclear | Not mentioned: “monitored using electrophysiology... intrinsic optical imaging” |
| Incomplete outcome data | Low risk | Complete sample size reported for all experiments with no missing data. |
| Selective outcome reporting | Low risk | All predefined primary outcomes were fully reported: “CSD number/latency/magnitude/MDA” |
| Other sources of bias | Low risk | shows no evidence of contamination, funder influence, or unit-of-analysis errors. |

Pusic AD *et al*. [24] (2015a)

| Evaluation Item | Risk Assessment | Supporting Evidence |
| --- | --- | --- |
| Sequence generation | Unclear | Not mentioned: “Wistar rats were obtained from Charles River; We prepared 350 μm hippocampal slice cultures from Wistar rat pups...” |
| Baseline characteristics | Unclear | Sex, weight range, and housing reported; specific physiological parameters (*e.g.*, detailed weight/age) not provided: “Control slice cultures; age-matched controls; Slices with CA3 field post-synaptic responses ≥3 mV were used” |
| Allocation concealment | Unclear | Not mentioned |
| Random housing | Unclear | Not mentioned: “All slices/animals maintained under identical conditions: maintained in serum-free medium, incubated under normal conditions.” |
| Performance bias | Unclear | Not mentioned |
| Blinding outcome assessment | Unclear | Not mentioned: “Images were acquired; Quantification of fluorescence intensity” |
| Detection bias | Unclear | Not mentioned: “Western blot analyses; densitometric quantification” |
| Incomplete outcome data | Low risk | Complete sample size reported for all experiments with no missing data: “all samples included: *” “n = 3 slices/group”, “n = 4 animals/group” |
| Selective outcome reporting | Low risk | All predefined primary outcomes were fully reported: “*e.g.*, EM, PCR, immunohistochemistry” |
| Other sources of bias | High risk | The authors (A.D.P., H.H.M., R.P.K.) report a patent pending enti-tled |

Grinberg YY *et al*. [25] (2013)

| Evaluation Item | Risk Assessment | Supporting Evidence |
| --- | --- | --- |
| Sequence generation | Unclear | Not mentioned: “some slices were placed in media supplemented with IGF-1” |
| Baseline characteristics | Unclear | No reporting of baseline characteristics like animal weight or slice vitality: “SD was induced...” |
| Allocation concealment | Unclear | Not mentioned |
| Random housing | Low risk | “The study utilized *ex vivo* brain slice cultures, without involving in vivo animal housing: ‘All electrophysiological and pharmacological manipulations were performed... in serum-free media’” |
| Performance bias | Unclear | Not mentioned: nNo mention of blinding during interventions, *e.g.*, IGF-1/TNF-α administration |
| Blinding outcome assessment | Unclear | Not mentioned: “3–5 confocal images were taken per slice” |
| Detection bias | Unclear | Not mentioned: “Blinding only for OS measurement, not specified for other outcomes like TNF-α or electrophysiology” |
| Incomplete outcome data | Low risk | Complete sample size reported for all experiments with no missing data: “A blinded investigator selected OS-labeled cell bodies...” |
| Selective outcome reporting | Low risk | All predefined primary outcomes were fully reported: “All described outcomes—OS, TNF-α, SD threshold” |
| Other sources of bias | High risk | “filed an international patent application (PCT/US2012/047683) entitled ‘Treatments for Migraine and Related Disorders’ on 20 July 2012 which partially involves development of IGF-1 as a therapeutic for migraine” |

Pusic KM *et al*. [26] (2019)

| Evaluation Item | Risk Assessment | Supporting Evidence |
| --- | --- | --- |
| Sequence generation | Unclear | Not mentioned: “Male Wistar rats (10–12 weeks old) were double-housed” |
| Baseline characteristics | Low risk | Uniform baseline conditions: “Male Wistar rats (10–12 weeks old); Untimed pregnant Wistar female rats...” |
| Allocation concealment | Unclear | Not mentioned |
| Random housing | Low risk | All animals were housed in the same environment, meeting the requirement for randomized housing: “double-housed; single-housed with Enviro-dri paper bedding and Nestlets” |
| Performance bias | Unclear | Not mentioned: “SD threshold determined; Quantification... by BCA assay” |
| Blinding outcome assessment | Unclear | Not mentioned: “Recording microelectrodes... positioned using WR 60 manipulators; Protein carbonyl levels... according to manufacturer’s protocol” |
| Detection bias | Unclear | Not mentioned |
| Incomplete outcome data | Low risk | Complete sample size reported for all experiments with no missing data: “n = 8/group” |
| Selective outcome reporting | Low risk | All predefined primary outcomes were fully reported: “SD threshold, iNOS, protein carbonyl detection” |
| Other sources of bias | Low risk | shows no evidence of contamination, funder influence, or unit-of-analysis errors. |

Pusic AD *et al*. [27] (2015b)

| Evaluation Item | Risk Assessment | Supporting Evidence |
| --- | --- | --- |
| Sequence generation | Unclear | Not mentioned: “Wistar rats were obtained from Charles River Laboratories”; “hippocampal slice cultures were made from the brains of P10 Wistar rats” |
| Baseline characteristics | Low risk | Uniform baseline conditions: “Wistar rats were obtained from Charles River Laboratories; hippocampal slice cultures were prepared from P10 pups; age-matched untreated cultures served as controls” |
| Allocation concealment | Unclear | Not mentioned |
| Random housing | Low risk | Uniform baseline conditions: “maintained under normal incubation conditions (36 ℃, 95% humidity and 5% carbon dioxide-balance air)” |
| Performance bias | Unclear | Not mentioned |
| Blinding outcome assessment | Unclear | Not mentioned |
| Detection bias | Unclear | Not mentioned: “For immunohistochemical staining... no mention of blinding (*e.g.*, Immunostaining... was accomplished as previously described” |
| Incomplete outcome data | Low risk | “Complete sample size reported for all experiments with no missing data: ‘n = 6–12/group’” |
| Selective outcome reporting | Low risk | All predefined primary outcomes were fully reported: “MBP, SDT, OS, GSH...” |
| Other sources of bias | High risk | The investigator holds patents related to the research findings, which may introduce conflict of interest bias: “The authors report patents pending... no other disclosures” |

Ackermann MA *et al*. [13] (2024)

| Evaluation Item | Risk Assessment | Supporting Evidence |
| --- | --- | --- |
| Sequence generation | Unclear | Not mentioned: “typically using 3–4 slices from each brain; each experimental paradigm was conducted on at least 3 different mice” |
| Baseline characteristics | Unclear | Only reported the age and sex of mice, without comparing baseline indicators between groups: adult male roGFP mice... average age was 5.8 ± 2.9 months |
| Allocation concealment | Unclear | Not mentioned |
| Random housing | Low risk | All sections were processed under identical conditions: “submerged in a storage chamber with oxygenated regular ACSF”, “heated to 35.5–36.0 ℃ and constantly supplied with fresh oxygenated ACSF” |
| Performance bias | Unclear | Not mentioned |
| Blinding outcome assessment | Unclear | Not mentioned: “ROIs (~20 × 40 μm) near the recording electrode” |
| Detection bias | Unclear | Not mentioned |
| Incomplete outcome data | Low risk | Complete sample size reported for all experiments with no missing data: only those slices were included... which presented reasonable optical and electrophysiological data |
| Selective outcome reporting | Low risk | All predefined primary outcomes were fully reported: “We analyzed SD-related ROS production... induced by K⁺ microinjection, O₂ withdrawal or mitochondrial uncoupling” |
| Other sources of bias | Low risk | shows no evidence of contamination, funder influence, or unit-of-analysis errors. |

Grinberg YY *et al*. [28] (2012)

| Evaluation Item | Risk Assessment | Supporting Evidence |
| --- | --- | --- |
| Sequence generation | Unclear | Not mentioned: “slice cultures were prepared from nine day old male or female pups of Wistar rats” |
| Baseline characteristics | Low risk | Uniform baseline conditions: prepared from nine day old male or female pups of Wistar rats, AND Slices with CA3 field post-synaptic potential responses >3 mV... were used” |
| Allocation concealment | Unclear | Not mentioned |
| Random housing | Low risk | Uniform baseline conditions: “slice cultures were maintained in serum-free media” |
| Performance bias | Unclear | Not mentioned |
| Blinding outcome assessment | Unclear | Not mentioned |
| Detection bias | Unclear | Not mentioned |
| Incomplete outcome data | Low risk | Complete sample size reported for all experiments with no missing data: “Fig. 1: n = 6–11; Fig. 2: n = 3–21” |
| Selective outcome reporting | Low risk | All predefined primary outcomes were fully reported: “We analyzed SD-related ROS production... induced by K⁺ microinjection, O₂ withdrawal or mitochondrial uncoupling” |
| Other sources of bias | Low risk | shows no evidence of contamination, funder influence, or unit-of-analysis errors. |

Won L *et al*. [29] (2020)

| Evaluation Item | Risk Assessment | Supporting Evidence |
| --- | --- | --- |
| Sequence generation | Unclear risk | Not mentioned: “Stated group allocation only, but method of random sequence generation not described: Rats were randomly divided into control and experimental groups” |
| Baseline characteristics | Unclear | Not mentioned: “Failed to formally compare inter-group baseline differences or perform statistical adjustments: Adult (250–450 g) male Wistar rats (n = 90 total)” and “All treatments and data analyses were completed under blinded conditions.” |
| Allocation concealment | Unclear risk | Not mentioned |
| Random housing | Low risk | Uniform baseline conditions: “Housing included use of static micro isolator cages... maintained in a 12-hour light-dark cycle with controlled humidity and temperature” |
| Performance bias | Low risk | c-fos labeled nuclei were manually counted by an observer blinded to experimental conditions.” |
| Blinding outcome assessment | Low risk | “All treatments and data analyses were completed under blinded conditions. |
| Detection bias | Low risk | imaging was performed ... under blinded conditions” AND “c-fos labeled nuclei were manually counted ... by an observer blinded to experimental conditions |
| Incomplete outcome data | Low risk | Complete sample size reported for all experiments with no missing data: “n = 5/group, n = 8/group at 0 hour” |
| Selective outcome reporting | Low risk | All predefined primary outcomes were fully reported: “MDA, CGRP, c-fos...” |
| Other sources of bias | Low risk | shows no evidence of contamination, funder influence, or unit-of-analysis errors. |

Malkov A *et al*. [14] (2014)

| Evaluation Item | Risk Assessment | Supporting Evidence |
| --- | --- | --- |
| Sequence generation | Unclear | Not mentioned: “P21–P56 adult Swiss male mice”, “5 mature (>P50) Swiss male mice” |
| Baseline characteristics | Low risk | Uniform baseline conditions: “P21–P56 adult Swiss male mice, saggital slices (350 μm, recover for 2 hours” |
| Allocation concealment | Unclear | Not mentioned. |
| Random housing | Low risk | Uniform baseline conditions: “slices recovered in continuously circulating ACSF”, “mice kept under saline to prevent drying” |
| Performance bias | Unclear | Not mentioned: “Tempol injected into ventricles” |
| Blinding outcome assessment | Unclear | Not mentioned: “recorded using...amplifier”, “images acquired using camera” |
| Detection bias | Unclear | Not mentioned: “measured in three to five regions of interest” |
| Incomplete outcome data | Low risk | Complete sample size reported for all experiments with no missing data: “14 out of 40 experiments (35%)”, “irreversible in 7 out of 14” |
| Selective outcome reporting | Low risk | All predefined primary outcomes were fully reported: All preset indicators (electrophysiological, metabolic, ROS fluorescence signals) were reported. Supplementary data (**Supplementary Figs. 1,2,3**) were mentioned but not shown in the main text, as is conventional. |
| Other sources of bias | Low risk | shows no evidence of contamination, funder influence, or unit-of-analysis errors. |

Viggiano A *et al*. [30] (2011)

| Evaluation Item | Risk Assessment | Supporting Evidence |
| --- | --- | --- |
| Sequence generation | Unclear | Not mentioned: “Rats were divided in the following groups: group 1 (5 rats)... group 2 (5 rats)... group 3 (5 rats)” |
| Baseline characteristics | Low risk | Uniform baseline conditions: “male Sprague-Dawley rats (250–300 g)... housed in pairs at controlled temperature (22 ± 1 ℃), and humidity (70%) with 12:12 h light-dark cycle” |
| Allocation concealment | Unclear | Not mentioned: “Rats were divided...” |
| Random housing | Low risk | Uniform baseline conditions: “housed in pairs at controlled temperature (22 ± 1 ℃)... 12:12 h light-dark cycle” |
| Performance bias | Unclear | Not mentioned: “KCl was applied... DHE injected... animals sacrificed” |
| Blinding outcome assessment | Unclear | Not mentioned: “Three sections were uniformly randomly sampled” |
| Detection bias | Unclear | Not mentioned |
| Incomplete outcome data | Low risk | Complete sample size reported for all experiments with no missing data: “Group 1 (5 rats)... Group 2 (5 rats)... Group 3 (5 rats)” |
| Selective outcome reporting | Low risk | All predefined primary outcomes were fully reported: “O₂, H₂O₂, SOD...” |
| Other sources of bias | Low risk | shows no evidence of contamination, funder influence, or unit-of-analysis errors. |

Shatillo A *et al*. [31] (2013)

| Evaluation Item | Risk Assessment | Supporting Evidence |
| --- | --- | --- |
| Sequence generation | Unclear | Not mentioned: “Wistar rats (6 weeks old, weighing 133–177 g) were used. Only mentions animal usage without specifying randomization methods for group allocation.” |
| Baseline characteristics | Unclear | Not mentioned: “Wistar rats (6 weeks old, weighing 133–177 g). No inter-group comparison data (*e.g.*, weight, sex) provided” |
| Allocation concealment | Unclear | Not mentioned: “Application of KCl to dura mater. No description of concealment methods (*e.g.*, sealed envelopes, coding systems) provided” |
| Random housing | Unclear | Not mentioned: “continuously superfused with oxygenated Krebs control solution” |
| Performance bias | Unclear | Not mentioned: “Application of hydrogen peroxide to meninges” (Abstract); “applied the oxidant H₂O₂” |
| Blinding outcome assessment | Unclear | Not mentioned: “frozen tissue samples were homogenized” |
| Detection bias | Unclear | Not mentioned. |
| Incomplete outcome data | Low risk | All animals included in the analysis: “Sample sizes (n-values) were explicitly reported for each group (*e.g.*, ‘n = 6 in CSD animals and n = 5 in sham control’, Fig. 2), with no mention of animal attrition or missing data.” |
| Selective outcome reporting | Low risk | All predefined primary outcomes were fully reported:  “- MDA: Methods (TBARS assay) → Results (CSD induced changes in lipid peroxidation)  - Electrical activity: Methods (Electrical activity) → Results (H₂O₂ increases electrical activity)  - CGRP: Methods (CGRP release assay) → Results (H₂O₂ triggers CGRP release).” |
| Other sources of bias | Low risk | shows no evidence of contamination, funder influence, or unit-of-analysis errors. |
